# Longitudinal associations between loneliness and outcomes of Common Mental Disorders (CMDs): A systematic review and meta-analysis

**DOI:** 10.1186/s12916-026-05252-6

**Published:** 2026-10-03

**Authors:** Theodora Stefanidou, Siying Li, Kinga Lagan, Gamze Evlat, Hannah Gray, Jasmine Harju-Seppänen, Amber Jarvis, Millie O’Sullivan, Shivangi Talwar, Gemma Lewis, Joshua E. J. Buckman, Alexandra Pitman, Sonia Johnson, Brynmor Lloyd-Evans

**Affiliations:** 1 Division of Psychiatry, University College London (UCL), London, UK; 2 Centre for Outcomes Research and Effectiveness (CORE), Research Department of Clinical, Educational & Health Psychology, University College London (UCL), London, UK; 3 North London NHS Foundation Trust, London, UK; 4 Institute of Psychiatry, Psychology & Neuroscience, King’s College London, London, UK; 5 Department of Psychology, Kingston University, London, UK; 6 Camden & Islington NHS Talking Therapies for anxiety and depression services, North London NHS Foundation Trust, London, UK

**Keywords:** Loneliness, Depression, Anxiety, Common mental disorders, Mental health, Longitudinal, Systematic review, Meta-analysis

## Abstract

**Background:**

Loneliness has been increasingly associated with poor mental health outcomes, yet its prognostic role in people with Common Mental Disorders (CMDs) remains unclear. This systematic review aimed to examine whether loneliness is longitudinally associated with mental health outcomes in individuals with CMDs, regardless of treatment status.

**Methods:**

We searched PsycINFO, MEDLINE, Embase and CINAHL from inception to October 2025 for longitudinal studies examining associations between baseline loneliness and CMD outcomes at follow-up. Eligible studies included adults aged 16+ with CMDs. We conducted a narrative synthesis and where appropriate, random-effects meta-analyses for binary depression outcomes reflecting unfavourable course (recurrence, persistence, or non-remission). Study quality was assessed using the Newcastle-Ottawa Scale, and certainty of evidence was evaluated using GRADE. The protocol was registered with PROSPERO (registration number: CRD42023410401).

**Results:**

We included 22 studies, of which the majority focused on depression-related outcomes (operationalised as symptom severity, recurrence, persistence, remission, and service utilisation). Our narrative synthesis showed consistent associations between higher baseline loneliness and poorer depression-related outcomes at follow-up in people with CMDs across clinical, community, and treatment settings. Meta-analysis of four studies (all in older adults with depression) showed that individuals reporting loneliness at baseline had over three times the odds of poor depression outcomes at follow-up compared with those not lonely (pooled adjusted OR 3.26, 95% CI [2.26–4.70], p < 0.001). This finding should be interpreted with caution, as only one of the four studies adjusted for baseline depressive symptom severity. The few studies capturing suicidal ideation, anxiety, and mixed CMD outcomes found limited and inconsistent evidence. The GRADE certainty of evidence was high for depression outcomes, low for suicidal ideation and anxiety outcomes, and very low for mixed CMD outcomes.

**Conclusions:**

Loneliness is consistently associated with poorer subsequent depression outcomes in people with CMDs, particularly depressive disorders. These findings suggest loneliness may represent an important prognostic factor in depression and warrant further evaluation in intervention studies. Further high-quality longitudinal research is needed to clarify whether—and under what conditions—loneliness affects outcomes for other CMDs and treatment effectiveness.

## Background

### Conceptualisations of loneliness

Loneliness is a distressing emotional state stemming from the subjectively experienced gap between one’s actual and desired social relations [1]. It is related to perceived social isolation and is associated with the quality of social relationships [2]. While often associated with social isolation, loneliness extends beyond physical solitude and should be distinguished from quantitative features of social support, such as objective social isolation and social network size [3].

The sociologist Weiss [4] suggested two primary dimensions of loneliness: social and emotional. According to this typology, social loneliness results from difficulties with social integration, and emotional loneliness is derived from the absence of a close intimate relationship. Expanding on Weiss’s framework, Cacioppo et al. [5] conceptualised loneliness as comprising three dimensions: intimate, relational, and collective loneliness. Intimate loneliness refers to the absence of a close emotional bond (partner, best friend), relational loneliness refers to the lack of quality friendships or infrequent contact with friends/family, and collective loneliness entails the lack of connection to wider social identities or communities (e.g., clubs, religious, or cultural groups). These models explain loneliness as a multifaceted phenomenon with diverse manifestations across contexts and individuals.

Psychometrically robust self-report measures of loneliness have been developed and used extensively in research. The UCLA Loneliness Scale, developed by Russell et al. [6], is one of the most widely used measures, assessing subjective feelings of loneliness and social isolation. Additionally, the De Jong Gierveld Loneliness Scale offers an assessment of loneliness which encompasses both emotional and social dimensions [7].

### Loneliness and common mental disorders

Loneliness is increasingly recognised as a critical public health issue with evidence of adverse effects on physical and mental health [2, 8, 9]. Chronic loneliness is associated with increased risk of various health problems, including cardiovascular disease, hypertension, weakened immune function, and elevated mortality rates [10]. Furthermore, loneliness is associated with negative mental health outcomes, including increased symptoms of Common Mental Disorders (CMDs) [5, 11], suicidal thoughts, and suicide attempt [12]. According to National Institute for Health and Care Excellence (NICE) clinical guidelines, CMDs include depression and anxiety disorders such as generalised anxiety disorder (GAD), panic disorder, mixed depression and anxiety, obsessive-compulsive disorder (OCD), post-traumatic stress disorder (PTSD), social anxiety disorder and phobias about specific things or situations [13].

Depression is a leading cause of disease burden globally [14, 15]. The bidirectional relationship between loneliness and depression is well established, with longitudinal studies finding that loneliness serves as both a risk factor for the onset of depressive symptoms and as a consequence of experiencing them [16, 17]. Similarly, anxiety disorders have been linked to experiences of loneliness [18]. A meta-analysis identified that social isolation—including loneliness— is closely associated with social anxiety disorder, although causality remained unclear due to limited longitudinal evidence [19]. Longitudinal findings from a general population sample suggest that while loneliness predicts later symptoms of depression, social anxiety, and paranoia, only social anxiety prospectively predicts subsequent loneliness, highlighting its key role in the maintenance of loneliness [20].

The relationship between loneliness and mental health extends beyond clinical outcomes, encompassing broader implications for individuals’ psychological well-being and quality of life [11]. Chronic loneliness has been associated with heightened levels of psychological distress, decreased self-esteem, and impaired interpersonal functioning, exacerbating individuals’ vulnerability to mental health disorders [21]. Loneliness has also been associated with maladaptive coping strategies, such as substance abuse and disordered eating behaviours [22].

Much of the existing longitudinal evidence has focused on loneliness as a risk factor for the onset of mental health problems in general population samples [17]. However, whether loneliness influences prognosis among individuals already experiencing CMDs is a distinct and clinically relevant question, with implications for understanding the course of CMDs and treatment outcomes. One prior systematic review examined whether loneliness and perceived social support predict poor outcomes among adults with mental health problems [23] and found some evidence that greater loneliness predicted poorer depression outcomes. However, the review included only two studies that directly assessed loneliness and just five of the 34 eligible studies involved long-term follow-up. While understanding both short- and long-term impacts of loneliness on mental health outcomes in clinical populations is important, longer-term follow-up helps clarify whether loneliness reflects a transient emotional state associated with short-term symptom exacerbation or a more sustained factor influencing the course of disorder. Finally, it remains unclear whether and how loneliness may affect the outcomes of psychological or other treatments and if people who are lonely at baseline experience poorer outcomes from mental health interventions.

Thus, this study aimed to address this important gap in knowledge by systematically reviewing the evidence on whether loneliness is associated with subsequent poor outcomes among people with CMDs. In particular, we focused on loneliness as the sole exposure of interest, and adults of all ages with CMDs as the specific population. Our primary review question was:

Is loneliness longitudinally associated with mental health outcomes among adults with CMDs?

As a secondary aim, we investigated whether being lonely is associated with poorer treatment outcomes; an important question with direct clinical relevance, especially if loneliness is not directly addressed in treatment.

The review follows guidance from the Centre for Reviews and Dissemination on undertaking reviews in health care and the Preferred Reporting Items for Systematic Reviews and Meta-analyses (PRISMA statement) [24, 25]. A PRISMA checklist for this review is provided in the Additional file: Appendix 1.

## Methods

### Protocol and registration

The review protocol was registered at PROSPERO international prospective register of systematic reviews at the Centre for Reviews and Dissemination, University of York (CRD42023410401) [26].

### Eligibility criteria

- Publication types: Peer-reviewed published papers, grey literature and preprints were eligible.
- Study types: All longitudinal studies, including both observational and intervention studies that reported a longitudinal association between loneliness at T1 and subsequent mental health outcomes at T2 were included in the review. All other types of studies such as cross-sectional studies, qualitative studies, systematic reviews, and meta-analyses were excluded. However, we considered any eligible studies that were included within relevant reviews or meta-analyses.
- Participants: Studies were included if their samples consisted of adults aged 16+ years with CMDs. For this review, we followed the NICE clinical guidance [13] that defines CMDs as depression and anxiety disorders such as GAD, panic disorder, mixed depression and anxiety, OCD,PTSD, social anxiety disorder as well as phobias about specific things or situations. The lower age threshold of 16 years was selected to reflect the structure of mental health services and epidemiological research in the UK and Europe. Our intention was to exclude child-only samples while allowing inclusion of late-adolescent participants receiving adult-oriented care. Studies including mixed adolescent/adult samples were eligible only where data for participants aged ≥16 years could be isolated. Clinical populations were included however diagnosis was made, including clinical diagnoses, structured assessment tools, self-reported assessment tools or use of relevant clinical service. Within general population samples, at least 50% of participants had to meet the CMD symptom threshold, as determined by clinical cut-offs specific to each screening tool or measure. If a study reported findings separately for those meeting CMD criteria, it was included, even if fewer than 50% of the total sample met CMD criteria. We excluded studies concerning samples of children under 16 years of age, people with drug and alcohol disorders only, people with a primary diagnosis of severe mental illness (SMI) (psychosis, bipolar disorder, depression with psychotic features, personality disorders), or CMDs secondary to or following the onset of neurological or neurodegenerative disorders/diseases (e.g., traumatic brain injuries, stroke, dementia, Parkinson’s disease), or populations with organic brain disorders.
- Exposure variable(s): loneliness at baseline (one time point) measured using any measure including validated quantitative scales (e.g., the De Jong Gierveld Loneliness Scale or the UCLA Loneliness Scale) and single-item measures of loneliness. Studies evaluating social isolation and related concepts but not loneliness were excluded.
- Comparator(s): Potential comparators included participants reporting either no loneliness or lower levels of loneliness.
- Outcomes: Studies were eligible if they assessed clinical or functional (e.g., social functioning) outcomes at follow-up. The main outcome of the review was CMD symptom severity, including measures of depression, anxiety, OCD, PTSD, and overall mood. We also included suicidal ideation only where it was explicitly measured and analysed as a distinct outcome. Although it is a symptom of depression, it is not synonymous with depressive severity or remission and may occur in other disorders. Suicidal ideation was therefore synthesised separately from depressive outcomes. This included measures assessing thoughts of harming oneself regardless of intent (e.g., PHQ-9 item 9), which would include some instruments that do not distinguish suicidal ideation from thoughts about non-suicidal self-harm. Eligible studies were required to assess CMD symptom severity at follow-up using validated instruments. In addition to CMD symptoms, secondary outcomes included well-being, global mental health, relapse following remission, admission to hospital or crisis services, quality of life, social functioning, and personal recovery. To be eligible, studies must have assessed these outcomes at follow-up using validated instruments. Relapse had to be measured through a recorded DSM or ICD diagnosis during outpatient or inpatient contact or using other reliable and validated tools. Hospital or crisis service admissions must have included voluntary or compulsory psychiatric hospitalisations, and/or use of crisis services. Overall functioning, well-being, quality of life, and personal recovery had to be assessed using validated instruments appropriate to each construct.

### Search strategy

We searched for relevant studies through four electronic databases: PsycINFO, MEDLINE, Embase and CINAHL from inception to March 2023, combining text words and subject headings (MeSH terms) for loneliness, CMDs and study design (see Additional file: Appendix 2). The search terms were adapted from key reviews: Wang et al. [23] and Surkalim et al. [27]. We consulted a specialist systematic reviewer in the CORE Unit (Centre for Outcomes and Research and Effectiveness) in the Department of Psychology and Language Sciences at University College London to ensure that our search strategy was comprehensive. The search was limited to humans, and no language or publication date restrictions were imposed.

The search was updated across all databases in May 2024 and in October 2025. We reran the original search strategy and applied an entry date filter, which retrieves articles added to the database since our last search, regardless of their publication date. Unlike a publication date filter that only captures newly published articles, the entry date filter ensures that all recently indexed articles are included in the results. We searched for additional studies and grey literature (non-peer reviewed papers) through Google Scholar by searching the first 10 pages of search results on similar terms to those used in the database searches. We also searched relevant preprint servers (medRxiv, PsyArXiv) for eligible unpublished studies.

Backward citation tracking was conducted by reviewing the reference lists of included studies to identify relevant papers. We reviewed the citations and made note of any records that were relevant to our review. We cross-checked Rayyan (a web-based systematic review management platform) to make sure the citations had not been screened already and then screened the remaining records. This process helped ensure comprehensive coverage of related literature in the review.

### Screening

Two members of the review team (TS, GE) piloted the selection strategy. All titles and abstracts were screened, with a random 26% of the records independently screened by two reviewers (TS, GE, MO, MN, JHS, ST, AJ, KL). All full texts were independently screened by two reviewers (ST, KL, JHS, GE, HG, AJ). Screening guides are provided in Additional file: Appendix 3. Any disagreements were resolved by consensus, and when necessary, by involving a third reviewer (BLE or SJ). Records were deduplicated in EndNote and then exported to Rayyan, where title and abstract as well as full-text screening were carried out. Non-English records were screened using translation tools (e.g., Google Translate) and, where appropriate, native speakers assisted with confirming eligibility and data extraction.

### Data extraction

A data extraction form in Microsoft Excel was developed by TS for study characteristics and outcome data. The extraction form included data from the included studies, such as first author and year of publication, country, study design, participants, setting, outcomes, method of analysis and results. The extraction form was piloted on 10% of included studies by two reviewers (TS, HG) and revised accordingly. Data were extracted by one reviewer (TS, HG, KL).

### Quality and certainty of evidence

The quality of included studies was assessed using the Newcastle-Ottawa Scale (NOS), which is designed for evaluating the quality of non-randomised studies, particularly cohort and case-control studies [28]. In the NOS, a ‘star system’ [28] has been developed in which a study is judged on the three main areas below:

1. Selection of participants (4 items): Evaluates how participants were chosen, whether inclusion/exclusion criteria were appropriate, and if groups were comparably selected.
2. Comparability of groups (1 item): Assesses whether the groups being compared are comparable on certain characteristics or controlling for confounding factors.
3. Outcome assessment (3 items): Looks at how outcomes were measured and whether the assessment methods were reliable and consistent.

For the purposes of this review, Randomised Controlled Trials (RCTs) were not analysed in terms of intervention effects. Instead, they were treated as prospective longitudinal cohort studies, examining associations between baseline loneliness and subsequent CMD outcomes within the trial samples. Randomisation and treatment allocation were not relevant to the exposure–outcome relationship addressed in our review question. We therefore applied the NOS to these studies to assess quality in relation to selection, comparability, and outcome domains relevant to the longitudinal associations of interest. This approach ensured consistency in quality appraisal across all included studies.

The NOS for cohort studies was operationalised to fit our research question (see Additional file: Appendix 4). Two review authors independently assessed the risk of bias in included studies (TS, HG). Disagreements between the two assessors were resolved through discussion with a third review author (BLE). Certainty of evidence for each outcome was assessed using the Grading of Recommendations Assessment, Development and Evaluation (GRADE) system [29]. We used the GRADE approach adapted for narrative synthesis and further adapted for the purposes of our review [30]. NOS scores were interpreted to inform judgments about the overall risk of bias in the GRADE assessment (see Additional file: Appendix 5).

### Data synthesis

A narrative synthesis was conducted informed by the Economic and Social Research Council guidance [31]. The characteristics of each included study were summarised, and findings were reported separately for each outcome: depression, suicidal ideation, anxiety and mixed outcomes. We classified studies by outcome category rather than by population in order to reduce heterogeneity and facilitate meta-analysis.

### Data analyses

Where at least three studies were sufficiently homogeneous in terms of exposure definition, outcome type, and statistical reporting, we conducted random-effects meta-analyses. The meta-analysis plan was developed by TS, SL, SJ and BLE following team discussions prior to analysis. TS extracted and prepared the data for meta-analysis, including exposure classification, outcome definition, effect estimates, confidence intervals, and covariate adjustment. SL cross-checked the extracted data and conducted all statistical analyses in R (version 4.0) using the package metafor for random-effects models.

We conducted the following meta-analyses for binary depression outcomes reflecting an unfavourable course (including recurrence, persistence, non-remission, chronic depression) grouped as poor mental health outcomes:

- Association of categorical loneliness with binary depression outcomes
- Association of continuous loneliness with binary depression outcomes

Separate meta-analyses were conducted for categorical and continuous loneliness measures because they estimate different underlying effect parameters (comparison of loneliness categories versus per-unit increase in loneliness). This approach was adopted to maximise the interpretability of pooled effect estimates, consistent with methodological guidance for quantitative synthesis of prognostic factor studies [32, 33]. An exploratory meta-analysis combining both measurement types was also conducted and is reported separately.

Log odds ratios were pooled using inverse-variance weighting. Random-effects models were fitted using Restricted Maximum Likelihood (REML) to estimate between-study variance (τ²). The pooled log(OR)s were then exponentiated into pooled ORs for interpretation. For studies reporting multiple categories of loneliness, a single contrast was selected comparing the highest loneliness category with the lowest/reference category. For studies reporting remission as the outcome, ORs were inverted prior to pooling to ensure consistency of direction. Adjusted estimates from the most fully specified models were prioritised. Where only a limited number of adjusted estimates was available, unadjusted estimates were included to maximise the available evidence and were interpreted cautiously. To clarify potential confounding by baseline symptom severity, we documented whether models contributing to pooled estimates adjusted for baseline depression severity. Heterogeneity was assessed using the I² statistic and by visual inspection of forest plots.

Continuous depression severity outcomes were not meta-analysed due to heterogeneity in modelling approaches (e.g., linear regression, mixed-effects models, trajectory analyses), variation in effect estimates (unstandardised coefficients, standardised βs), and insufficient reporting of parameters required for transformation to a standardised effect size. These findings were synthesised narratively.

Based on study characteristics and following team discussions, we distinguished three settings according to recruitment source:

A) General population samples (people recruited from the community not through mental health services; may or may not include individuals receiving any form of care)
B) Populations recruited from mental health services (e.g., community mental health teams, but without a clearly defined specific intervention being delivered across the sample)
C) Populations receiving a specific clinical treatment (e.g., RCTs or other intervention studies).

We examined whether the association between loneliness and CMD outcomes varied within and between these settings.

## Results

### Study selection

**Fig. 1:**
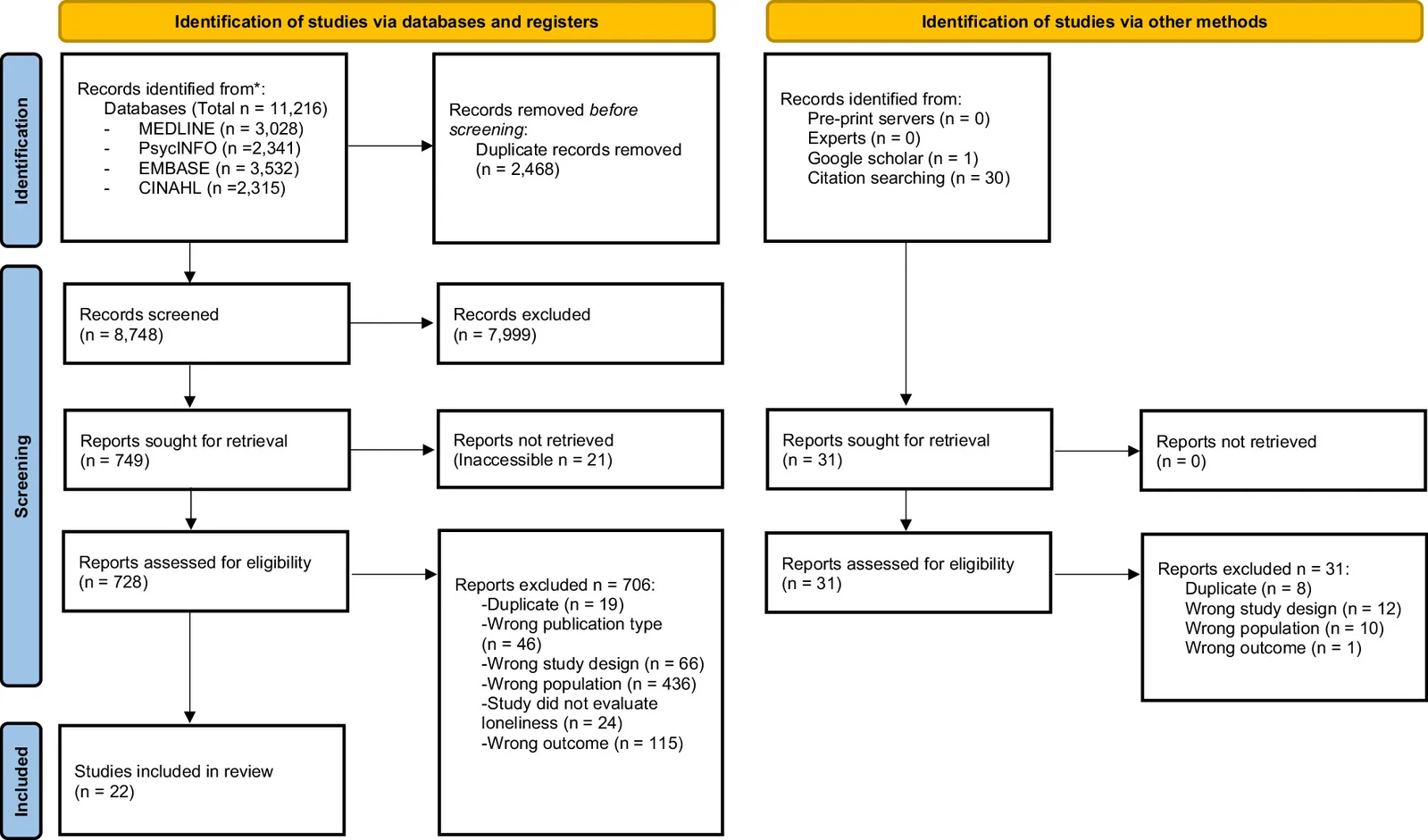
PRISMA 2020 flow diagram

The bibliographic database search yielded 11,216 citations. Another 31 records were identified through Google Scholar and citation tracking. After removing duplicates, 8,748 titles and abstracts were screened, and 728 full-text articles were assessed for eligibility. Twenty-two studies met the inclusion criteria [34–55]. The study selection process, including reasons for exclusion, is detailed in the PRISMA flow diagram [25] (Fig. 1).

Six additional studies were initially considered for inclusion as they examined associations between loneliness and CMD outcomes [56–61]. However, during the data extraction phase, it became evident that the reported associations did not meet the review’s prespecified longitudinal inclusion criteria. Most examined cross-sectional loneliness–CMD associations or concurrent changes in loneliness and CMD outcomes, while the remaining longitudinal analyses did not provide an eligible association between loneliness measured at an earlier time point and a subsequent CMD outcome. A summary of these ‘near misses’ is provided in Additional file: Appendix 6.

### Study characteristics

The characteristics of the 22 included studies are presented in Table 1. All included studies were peer-reviewed journal articles published between 2010 and 2025. Twenty-one studies were published in English, and one study (Ortiz et al. [49]) was published in Spanish. The studies were conducted in the Netherlands (n = 9), the United States of America (USA) (n = 6), Canada (n = 2), Australia (n = 1), Spain (n = 1), Brazil (n = 1), Mexico (n = 1) and the United Kingdom (UK) (n = 1). All included studies were longitudinal in design. Seventeen were observational cohort studies (both prospective and retrospective), and five were intervention trials.

**Table 1:** Characteristics of included studies (N = 22)

Author(s), Year	Country	Study design	Sample size N	Population	Setting	CMD status at baseline		CMD measure	Age Mean (SD), range	Gender N (%)	Ethnicity N (%)	Loneliness measure	Follow-up duration
A. General population (care unknown) (n = 7)													
Antonelli-Salgado et al., 2021 [34]	Brazil	Prospective cohort study	1,674	General population (9.3% screened positive for depression and 69.2% for anxiety)	Community – online survey	Anxiety (Primary); Depression (Secondary)		GAD-7; PHQ-9	38.6 (14.1), 18–75 years	Female: 1448 (86.5%) Male: 226 (13.5%)	NR	UCLA-3	1 month
Brown et al., 2023 [35]	USA	Longitudinal daily diary analysis	211	Community adults with elevated levels of depression and/or social anxiety	Community – recruited via social media and online platforms	Social anxiety and/or depressive symptoms		Mini Social Phobia Inventory; PHQ-9	34.32 (15.21)	Female: 81.0% Male: 17.5% Other: 1.4%	White/European American: 73.0% Hispanic/Latino: 21.3% Asian/Pacific Islander: 11.4% Black/African American: 7.6% Arab/Middle Eastern: 2.4% Other: 1.9%	UCLA-3 (adapted with additional item)	1.5 months
Gabarrell‐Pascuet et al., 2022 [37]	Spain	Prospective cohort	404	50+ adults with MDD	Community – nationally representative sample of noninstitutionalized adults in Spain	MDD		CIDI 3.0	65.75 (10.30), 50+ years	Female: 294 (72.77%) Male: 110 (27.23%)	NR	UCLA-3 (Spanish version)	3–4 years (Wave 2), 6–7 years (Wave 3)
MacNeil et al., 2023* [45]	Canada	Longitudinal cohort study	770	Older adults with asthma with a history of depression (CLSA)	Community – national stratified random sample	Depression		CES-D-10	60.7 (8.8)	Female: 532 (69.1%) Male: 238 (30.9%)	NR	Single-item loneliness question	Approximately 6 months (COVID Spring 2020 to COVID Autumn 2020)
MacNeil et al., 2024* [46]	Canada	Longitudinal cohort study	938	Older adults with migraine with a history of depression (CLSA)	Community – national stratified random sample	Depression		CES-D-10 > = 10 and a self-report single question of a previous diagnosis of depression	61.01 (8.80)	Female: 753 (80.3%) Male: 185 (19.7%)	NR	Single-item loneliness question	Approximately 6 months (COVID Spring 2020 to COVID Autumn 2020)
Nuyen et al., 2020 [48]	Netherlands	Longitudinal cohort study	4,007 (CMD subgroup n = 472)	Adults participating in the Netherlands Mental Health Survey and Incidence Study-2	Community	Severe CMD; Mild–Moderate CMD		CIDI	Total sample NR (age range: 18–64 years) CMD subgroup 42.9 (SE 0.95)	NR	NR	De Jong Gierveld Loneliness Scale	3 years
Ortiz et al., 2017* [49]	Mexico	Prospective cohort study	1,512 (Chronic depression subgroup n = 633)	Mexican older adults with chronic depression	Community households	Depression		Diagnosed using multiple criteria: - GMS-AGECAT for depressive syndrome - ICD-10 for depressive episode - DSM-IV for major depression - EURO-D scale for subsyndromal depression	Not reported for chronic depression group specifically; total sample age range: 65+ years (categorized as 65–69, 70–74, 75–79, 80 + ); mean age not provided	Chronic depression group: Women: 478 (75.5%) Men: 155 (24.5%)	NR	Single question from Geriatric Mental State inventory: “Do you feel lonely?” (yes/no response)	3 years
B. Using mental health services (n = 10)													
Chen et al., 2020 [36]	USA	Longitudinal cohort study	262	Veterans with depression	Primary care and other services at a Veterans Health Administration (VHA) facility	Depression	PHQ-2; PHQ-8		55.6 (13.8)	Male: 216 (82.4%), Female: 46 (17.6%)	White: 198 (75.6%) Black: 17 (6.5%) Latino: 15 (5.7%) AI/AN (American Indian/Alaska Native): 22 (8.4%) Other: 10 (3.8%)	NIH Toolbox Adult Social Relationship Scales	1 year
Holvast et al., 2015* [40]	Netherlands	Prospective cohort study	285	Older adults with late-life depression (NESDO)	Mental health care institutes, ambulatory and clinical mental health care, and general practices	Major depression, dysthymia, minor depression	CIDI		70.2 (64.5–76.0), ≥60 years	Male: 98 (34.4%)	NR	De Jong Gierveld Loneliness Scale	2 years
Jeuring et al., 2018* [41]	Netherlands	Prospective cohort study	510 (Depressed group n = 378, non-depressed group n = 132)	Older adults with late-life depression (NESDO)	Mental health care institutes, ambulatory and clinical mental health care, and general practice	Major depression, dysthymia, minor depression	CIDI		Depressed group: 69.0 (6.5), ≥60 years	Depressed group: Female: 137 (68.2%)	NR	Rasch-Type Loneliness Scale (RTLS)	6 years
Kivelä et al., 2019 [43]	Netherlands	Longitudinal cohort study	2,130 (Suicidal ideators subgroup n = 230)	Adults aged 18–65 years (NESDA)	Clinical sample – recruited from community and health care settings. Some were on medication.	Depression and anxiety disorders; suicidal ideation	CIDI; BSSI		Baseline suicidal ideators: 41.4 (12.0), 18–65 years	Baseline suicidal ideators: Female: 153 (67%)	NR (223 (97%) were Dutch)	De Jong Gierveld Loneliness Scale	2, 4, 6 and 9 years
Maarsingh et al., 2018* [44]	Netherlands	Multicenter cohort study	467	Older adults with MDD (NESDO, NESDA)	Community, primary care practices and mental health care organisations	MDD	CIDI		NESDO: 70.4, 60–90 years NESDA: 56.8, 50–66 years	NESDO Female: 178 (65.9%) NESDA Female: 135 (68.5%)	NR	De Jong Gierveld Loneliness Scale	2 years
Parmar et al., 2024 [50]	UK	Retrospective cohort study	26,737 (CMD subgroups n = 5,494 (4,056 with depression + 1,438 with anxiety))	Patients receiving mental healthcare	South London and Maudsley NHS Foundation Trust (SLaM) mental health provider	Depression (F32-F33) Anxiety (F40-F42)	ICD-10 codes		For the entire cohort (N = 26,737): Not recorded as lonely (n = 22,254): 46.4 (18.4) Recorded as lonely (n = 4,483): 47.9 (18.7)	For the entire cohort (N = 26,737): Not recorded as lonely (n = 22,254): female 10,497 (47.2%), Male 11,757 (52.8%). Recorded as lonely (n = 4,483): Female 2,501 (55.8%), Male 1,981 (44.2%)	For the entire cohort (N = 26,737): Not recorded as lonely (n = 22,254): White: 14,222 (63.9%) Asian: 1,275 (5.7%) Black: 4,688 (21.1%) Mixed: 541 (2.4%) Other: 1,399 (6.3%) Recorded as lonely (n = 4,483): White: 2,631 (58.7%) Asian: 292 (6.5%) Black: 1,231 (27.5%) Mixed: 116 (2.6%) Other: 210 (4.7%)	Electronic Health Records– NLP algorithm created to identify terms used by clinicians in notes that would identify patients as lonely or not lonely	7.5 years
Schaakxs et al., 2018 [51]	Netherlands	Cohort study	1,042	Adults aged 18–88 years with MDD (NESDO, NESDA)	Community, primary care practices and mental health care organisations	MDD	CIDI		18–88 years means by age group: 18–29 years: 24.11 (3.31) 30–39 years: 34.43 (2.92) 40–49 years: 44.47 (2.80) 50–59 years: 54.12 (2.82) 60–69 years: 63.61 (2.81) ≥70 years: 77.11 (5.15)	18–29 years: Female 130 (76%) 30–39 years: Female 130 (71%) 40–49 years: Female 141 (62%) 50–59 years: Female 122 (63%) 60–69 years: Female 101 (60%) ≥70 years: Female 66 (67%)	NR	Loneliness and Affiliation Scale (11-item)	2 years
van Beljouw et al., 2010 [52]	Netherlands	Longitudinal cohort study	594	Primary care patients with anxiety or depressive disorders (NESDA)	General practices (some respondents were receiving mental health support)	Anxiety disorders, depressive disorders	CIDI		45.7 (11.9), 18–65 years	423 female (71.2%)	NR	De Jong Gierveld Loneliness Scale	1 year
van den Brink et al., 2018* [53]	Netherlands	Naturalistic cohort	1,181	Adults with MDD (NESDO, NESDA)	Community, primary care practices and mental health care organisations	MDD	DSM-IV		Remission: 47.3 (16.8) Non-remission: 48.9 (16.4) 18–90 years	Remission n = 697: Female: 66.1% Non-remission n = 484: Female: 66.3%	NR	De Jong Gierveld Loneliness Scale	2 years
Zuidersma et al., 2025 [55]	Netherlands	Longitudinal cohort study	378	Depressed older adults (NESDO)	Mental healthcare facilities and general practitioners	Major depression, dysthymia, minor depression	CIDI		70.7 (SD: 7.4, range: 60–93)	Female: 250 (66.1%)	NR	Loneliness and Affiliation Scale	6 years
C. Receiving a specified clinical treatment (treatment response studies) (n = 5)													
Hassouneh et al., 2013 [38]	USA	Community-based participatory intervention, efficacy trial (analysed as a prospective cohort study)	80	Women with physical disabilities and depression	Community – Recruited from Centers for Independent Living, local disability service organizations, and other community settings	Depression	CES-D		51.81 (10.44)	Female: 80 (100%)	African American/black: 2 (2.53%) Unknown: 3 (3.8%) Asian: 1 (1.27%) Hispanic: 2 (2.53%) Multiracial: 9 (11.39%) White: 60 (75.95%) Native American: 2 (2.53%)	UCLA-LS-20	Post-intervention (14 weeks), 6 weeks and 3 months post-intervention
Hirshberg et al., 2025 [39]	USA	RCT (analysed as a prospective cohort study)	662	Adults working in education systems in Wisconsin (79.9% reported clinical levels of anxiety or depressive symptoms)	Community – recruited through web postings and newsletters	Depression and anxiety	Patient-Reported Outcomes Measurement Information System Inventory (PROMIS Anxiety & Depression scales)		HMP group (n = 344) <20: 0 (0.0%) 20–30: 55 (16.0%) 30–40: 105 (30.5%) 40–50: 103 (29.9%) 50–60: 61 (17.7%) >60: 20 (5.8%) WLC group (n = 318) <20: 1 (0.2%) 20–30: 46 (14.5%) 30–40: 90 (28.3%) 40–50: 93 (29.3%) 50–60: 79 (24.8%) >60: 9 (2.8%)	HMP group (n = 344): Female 302 (86.9%), Male 42 (12.2%), Non-binary 1 (0.3%) WLC group (n = 318): Female 281 (88.4%), Male 37 (11.65%), Non-binary 0 (0.0%)	HMP group (n = 344) American Indian/Alaskan Native: 5 (1.5%); Asian/Pacific Islander: 9 (2.6%); Black/African American: 11 (3.2%); Hispanic/Latino: 15 (4.4%); White/Caucasian: 315 (91.6%) WLC group (n = 318) American Indian/Alaskan Native: 4 (1.3%); Asian/Pacific Islander: 4 (1.3%); Black/African American: 15 (4.7%); Hispanic/Latino: 17 (5.3%); White/Caucasian: 287 (90.3%).	NIH Toolbox Loneliness Questionnaire (5-item)	3 months (16 weeks post-baseline)
Joo et al., 2025 [42]	USA	RCT (analysed as a prospective cohort study)	134	Older adults (aged 50 + ) with depressive symptoms (PHQ-9 ≥ 5)	Community	Depression (mean PHQ-9 10.6 (SD 4.78))	PHQ-9		Median (range): 69.5 (51.0–95.0)	Female: 113 (84.3%) Male: 21 (15.7%)	American Indian or Alaska Native: 1 (0.7%) Asian: 1 (0.7%) Black: 70 (52.2%) White: 55 (41.0%) Multiracial: 6 (4.5%) Other: 1 (0.7%) Hispanic: 3 (2.2%)	Revised UCLA Loneliness Scale (20-item R-UCLA)	Postintervention, 3, 6, 9, and 12 months
McGillivray et al., 2023 [47]	Australia	RCT (analysed as a prospective cohort study)	455	Young adults aged 18–25 with suicidal ideation and depressive symptoms	Recruited from the community	Suicidal ideation; depression (Mean PHQ-9 score 17.15 (SD 5.64))	SIDAS; PHQ-9		NR (age range: 18–25 years)	NR	NR	Three-Item Loneliness Scale (TILS)	Post-intervention (6 weeks) and 3 months post-intervention
Zech et al., 2025 [54]	USA	RCT (analysed as a prospective cohort study)	201	Adults with elevated social anxiety symptoms (SPIN ≥ 30)	Community – online via advertisements on Facebook and Instagram	Social anxiety	Social Phobia Inventory (SPIN)		SB: 28.14 (10.35) UB: 29.76 (12.77)	SB: Man: 15 (15.2%) Woman: 74 (74.7%) Other: 10 (10.1%) UB: Man: 17 (16.7%) Woman: 77 (75.5%) Other: 8 (7.9%)	SB (n = 99) Race: White 72 (72.7%); Black or African American 14 (14.1%); Asian or Pacific Islander 14 (14.1%); Native American/Alaskan Native 5 (5.1%); Other 3 (3.0%). Ethnicity: Hispanic or Latino/a 19 (19.2%); Not Hispanic or Latino/a 80 (80.8%). UB (n = 102) Race: White 65 (63.7%); Black or African American 11 (10.8%); Asian or Pacific Islander 28 (27.5%); Native American/Alaskan Native 1 (1.0%); Other 4 (3.9%). Ethnicity: Hispanic or Latino/a 17 (16.7%); Not Hispanic or Latino/a 85 (83.3%).	UCLA Loneliness Scale – Short Form (ULS-8)	8 weeks

*Included in meta-analyses.

** BSSI: Beck Scale for Suicide Ideation; CES-D: Centre for Epidemiologic Studies Depression Scale; CES-D-10: Short form of the Centre for Epidemiologic Studies Depression Scale; CIDI 3.0: Composite International Diagnostic Interview; CLSA: Canadian Longitudinal Study on Aging; DSM-IV: Diagnostic and Statistical Manual of Mental Disorders, Fourth Edition; EURO-D: European Depression Scale; GAD-7: Generalized Anxiety Disorder-7; GMS-AGECAT: Geriatric Mental State – Automated Geriatric Examination for Computer Assisted Taxonomy; HMP: Healthy Minds Program; ICD-10: International Classification of Diseases, Tenth Revision; MDD: Major Depressive Disorder; NESDA: Netherlands Study of Depression and Anxiety; NESDO: Netherlands Study of Depression in Older Persons; NLP: Natural Language Processing; NR: Not reported; PHQ-9: Patient Health Questionnaire-9; PROMIS: Patient-Reported Outcomes Measurement Information System; RTLS: Rasch-Type Loneliness Scale; SB: Safety Behaviour; SIDAS: Suicidal Ideation Attributes Scale; SPIN: Social Phobia Inventory; TILS: Three-Item Loneliness Scale; UB: Unhealthy behaviour; UCLA-3: University of California, Los Angeles Loneliness Scale (3-item version); ULS-8: UCLA Loneliness Scale – 8-item version; WLC: Wait-list control.

Studies covered a range of CMDs, with fourteen studies focusing on individuals with depression [36–38, 40–42, 44–47, 49, 51, 53, 55], five on mixed depression and anxiety [35, 39, 43, 50, 52], two on anxiety [34, 54] and one on CMDs more broadly [48]. CMD status at baseline was identified using structured diagnostic interviews (most commonly the Composite International Diagnostic Interview, CIDI), validated symptom measures and screening thresholds (e.g., PHQ-9, GAD-7, CES-D/CES-D-10, PROMIS). The included studies comprised both clinical populations with CMDs and general population samples with elevated symptoms of CMDs that met screening tool thresholds. Nine of the included studies (41%) focused on older adults ( ≥ 50 years), particularly those analysing data from the Netherlands Study of Depression in Older Persons (NESDO) and the Canadian Longitudinal Study on Aging (CLSA) cohorts [37, 40–42, 44–46, 49, 55]. Overall sample sizes ranged from 80 to 26,737 participants (CMD subgroups: 80 to 5494).

Most studies assessed loneliness using validated scales, with the UCLA Loneliness Scale and the De Jong Gierveld Loneliness Scale most commonly used. Three studies used a single-item question [45, 46, 49], and another used Natural Language Processing (NLP) algorithms to capture language in health records indicative of loneliness and distinguish between lonely and non-lonely patients [50]. Follow-up durations varied from one month to nine years: nine studies had follow-ups of less than one year, six ranged from one to two years, and seven followed participants for over two years. Of the 22 included studies, four examined loneliness as a mediator of CMD outcomes [35, 37, 39, 42].

Findings are presented by outcome: depression, suicidal ideation, anxiety, and mixed CMD outcomes. Four studies contributed results across multiple outcome categories and are referenced in more than one section [36, 47, 50, 52]. Three treatment settings were distinguished: A. General population (care status unknown) (n = 7); B. Using mental health services (n = 10); C. Receiving a specified clinical treatment (n = 5).

#### Methodological quality

Regarding quality assessment, 13 studies (59%) were rated as high quality, and 9 studies (41%) were rated as moderate quality according to the NOS criteria. Common limitations included high attrition rates, lack of detail on follow-up procedures, limited adjustment for confounders and inadequate description of comparison groups. GRADE certainty of evidence ratings were high for depression outcomes, low for suicidal ideation and anxiety outcomes, and very low for mixed CMD outcomes. Further details of the quality assessments and GRADE ratings are provided in Additional file (see Appendix 4: Table S1 and Appendix 5: Table S2).

### Loneliness and depression outcomes

Fifteen studies examined the association between baseline loneliness and depression outcomes at follow-up (see Table 2) [36–38, 40–42, 44–47, 49–53]. Of these, thirteen studies included samples of individuals with depression only [36–38, 40–42, 44–47, 49, 51, 53], while two studies involved mixed samples of participants with depression and anxiety [50, 52]. Studies were categorised by setting: general population (care status unknown, n = 4), mental health services (n = 8), and specific clinical treatment (n = 3). There was no observed relationship between setting and the statistical significance of findings.

**Table 2:** Study findings for depression outcomes (N = 15)

Author(s), Year	Setting	Main CMD outcome(s)	Main CMD outcome measure(s)	Adjusted for	Statistical methods	Results of the longitudinal association of baseline loneliness with CMD outcomes at follow-up (Effect size, 95% CI, P-value)	Intervention (if applicable)	Conclusions	NOS score
A. General Population (care unknown) (n = 4)									
Gabarrell‐Pascuet et al., 2022 [37]	Community - nationally representative sample of noninstitutionalized adults in Spain	Recurrent episodes of MDD	CIDI 3.0	Sex, age, partner status, educational level, and general health.	Cross‐lagged and autoregressive longitudinal associations using structural equation modelling	Adjusted: Lower social support at T0 (baseline) was associated with higher subsequent levels of loneliness at T1 (3 years) (Coef = −0.155, 95% CI [−0.249 to −0.061], p < 0.05), which in turn was associated with an increase in MDD recurrence at T2 (7 years) (Coef = 0.048, 95% CI [0.027–0.068], p < 0.05).	N/A	Loneliness was found to be longitudinally associated with lower levels of social support, and loneliness also was longitudinally associated with the recurrence of MDD. Therefore, loneliness is a mediator in the association between social support and MDD recurrence.	7 stars
MacNeil et al., 2023 [45]	Community - national stratified random sample	Recurrence of depression	CES-D-10	Age, sex, marital status, income, immigration status, visible minority status, education, household income, dwelling ownership, total saving, satisfaction with income, BMI, Chronic Pain, Multimorbidity, Adverse childhood experiences, Feel that lack companionship, Feel left out, Feel isolated from other, Church activities, Pray at home, COVID-related stressors	Multivariate Logistic Regression	Adjusted: Among older adults with asthma and a pre-pandemic history of depression, those who often felt lonely during the first few months of the COVID-19 pandemic were significantly more likely to have recurrent depressive symptoms at T1 (Autumn 2020 wave) (OR = 2.28, 95% CI [1.54–3.37], p < 0.001). Nagelkerke R² = 0.257	N/A	During the COVID-19 pandemic, loneliness significantly increased the risk of recurrent depression in older adults with asthma with a prior history of depression.	6 stars
MacNeil et al., 2024 [46]	Community - national stratified random sample	Recurrence of depression (depressed/not depressed based on CES-D-10 screening)	CES-D-10	Age, sex, marital status, immigration status, visible minority status, education, household income, Dwelling ownership, total saving, satisfaction with income, BMI, Chronic Pain, Multimorbidity, Adverse childhood experiences, Feel that lack companionship, feel left out, feel isolated from other, Church activities, Pray at home, COVID-related stressors	Multivariate Logistic Regression	Adjusted: Among older adults with migraine and a pre-pandemic history of depression, those who often felt lonely during the first few months of the COVID-19 pandemic at T0 (Spring 2020 wave) were significantly more likely to have recurrent depressive symptoms at T1 (Autumn 2020 wave) (OR = 3.00, 95% CI [2.07–4.34], p = 0.006; Nagelkerke R² = 0.288).	N/A	Older adults with migraine with a history of depression who often felt lonely during the first few months of the COVID-19 pandemic, were significantly more likely to have recurrent depressive symptoms at the autumn wave.	6 stars
Ortiz et al., 2017 [49]	Community households	Persistence of depression	GMS-AGECAT ICD-10 DSM-IV EURO-D scale	Sociodemographic (sex, education, marital status, occupation), environmental (urban/rural residence, food insecurity), psychosocial (contact with friends, life satisfaction, happiness), health (lifestyle, self-rated health, history of depression, number of limiting physical illnesses)	Multinomial logistic regression	Overall model (in participants with depression at baseline): Adjusted: Those who reported feeling lonely at baseline had approximately 4.63 times higher odds of experiencing persistent depression (depression present at both baseline and 3-year follow-up) compared to those who did not feel lonely (no depression reference group) (OR = 4.63, 95% CI [3.34–6.44], p < 0.05, exact p-value not reported).	N/A	For older adults with depression at baseline (chronic depression group), baseline loneliness was strongly associated with persistent depression at follow-up, indicating that those reporting loneliness were 4.63 times more likely to have persistent depression than no depression, adjusting for confounders.	7 stars
B. Using mental health services (n = 8)									
Chen et al., 2020 [36]	Primary care and other services at a Veterans Health Administration (VHA) facility	Depression severity Change in mental health visits (in-person, outpatient individual and group visits, and phone visits)	PHQ-9 Administrative medical record data	Age, gender, race/ethnicity, AUDIT-C screening score, PTSD screening score, medical comorbidity score, financial hardship, other social connectedness variables	Multivariable regression analysis	Adjusted: Higher baseline loneliness was associated with decreased depression severity from T0 to T1 (1 year) (B = −1.55, 95% CI [−2.53 to −0.56], p < 0.01). No significant associations between loneliness and change in mental health visits (B = −0.51, 95% CI [−3.40 to 2.39], p > 0.05).	N/A	Unexpectedly, greater loneliness at baseline was associated with greater improvement in depression symptoms over one year in a sample of veterans with depression. Baseline loneliness was not associated with change in mental health visits over one year.	7 stars
Holvast et al., 2015 [40]	Mental health care institutes, ambulatory and clinical mental health care, and general practices	Depression severity, depression remission	IDS-SR (severity), CIDI (remission)	Social network size, age, gender, marital status, education level, neuroticism, cognitive functioning, pain intensity, disability, number of chronic diseases, and setting, baseline symptom severity	Linear and logistic regression analyses, Multiple imputation by chained equations	Adjusted: Higher levels of loneliness at baseline (T0) were significantly associated with more severe depression at T1 (2 years) (B = 0.61, 95% CI [0.12–1.11], p = 0.02). The effect of loneliness could not solely be attributed to one of the two loneliness subscales; both emotional loneliness (beta = 0.76, 95% CI [0.004–1.52]) and social loneliness (beta = 0.99, 95% CI [0.07–1.92]) were associated with depression severity at follow-up. Adjusted: The very severely lonely respondents were less likely to achieve remission from depression compared with the non-lonely respondents at 2-year follow-up (OR = 0.25, 95% CI [0.08–0.80], p = 0.02). Moderate and severe loneliness were not statistically significant after adjustment.	N/A	Loneliness significantly affects the prognosis of late-life depression, with higher loneliness levels associated with worse depression outcomes.	9 stars
Jeuring et al., 2018 [41]	Mental health care institutes, ambulatory and clinical mental health care, and general practice	Depression severity; Depression course	Inventory of Depressive Symptomatology (IDS); CIDI	Generally: Gender, age, education, depression-related clinical factors including baseline depression severity, Health and lifestyle factors and psychological and social factors Severity outcome - final mixed model: Age of onset of depression, baseline IDS severity, anxiety severity, apathy, chronic diseases, functional limitations, chronic pain, body mass index, neuroticism, loneliness (all significant from group-wise models)	Bivariate multinomial logistic regression; Multivariate Linear Mixed Models	The paper does not provide adjusted ORs for remission categories. Unadjusted: Higher loneliness at T0 (baseline) was associated with chronic or recurrent depression as compared to full remission at T2 (6 years) (OR = 1.26, 95% CI [1.11–1.42], p < 0.001). Compared to full remission, partial remission was also associated with baseline loneliness (OR = 1.20, 95% CI [1.08–1.34], p = 0.001). Compared to partial remission, loneliness at T0 (baseline) was not associated with a chronic/recurrent (unfavourable) course of depression at T2 (6 years) (OR = 1.05, 95% CI [0.94–1.16], p = 0.39). Adjusted: In the group-wise model for psychological factors, baseline loneliness (T0) was associated with higher levels of depression severity at T2 (6 years) (β = 0.70 (SE = 0.20), p = 0.001). In the final model containing all prognostic factors, loneliness at T0 (baseline) was associated with higher levels of depression severity at T2 (6 years) (β = 0.39 (SE = 0.18), p = 0.036).	N/A	Loneliness was a significant factor associated with both the course and outcomes of late-life depression.	7 stars
Maarsingh et al., 2018 [44]	Community, primary care practices and mental health care organisations	Presence/absence of MDD at 2-year follow-up	CIDI	Age, onset of depression, severity of depression, anxiety symptoms, comorbid anxiety disorder, fatigue	Multivariable Logistic Regression	Adjusted: Higher levels of loneliness at T0 (baseline) were associated with the presence of MDD at T1 (2 years) (OR = 1.12, 95% CI [1.03–1.23], p = 0.007).	N/A	Loneliness was found to be an independent predictor of MDD after 2 years. Higher loneliness at baseline is associated with an increase in the odds of an unfavourable course of MDD.	8 stars
Parmar et al., 2024 [50]	SLaM Mental Healthcare Provider	Crisis episode*** Emergency presentation*** Number of days active in SLaM per year*** Number of face-to-face contacts per year***	Electronic health record data	Number of documents available to review, sociodemographic variables, Psychotropic medications, Psychiatric diagnoses, Physical health conditions and medications for physical health	Cox proportional hazard models; Poisson regression analyses	Adjusted Models: Crisis episode**:** Recorded loneliness at baseline in patients with depression was not significantly associated with an increased risk of crisis episodes at T1 (7.5 years follow-up) (HR = 0.95, 95% CI [0.74–1.24], p = 0.72). Emergency presentation**:** Recorded loneliness at baseline in patients with depression was significantly associated with higher risk of emergency presentations at T1 (7.5 years follow-up) (HR = 1.32, 95% CI [1.08–1.62], p = 0.01). Number of days active in SLaM and number of face-to-face contacts per year**:** Recorded loneliness at baseline in patients with depression was significantly associated with more days active per year (IRR = 1.12, 95% CI [1.11–1.13], p < 0.001) and more face-to-face contacts per year at T1 (7.5 years follow-up) (IRR = 1.41, 95% CI [1.36–1.47], p < 0.001).	N/A	In patients with depression receiving mental healthcare, recorded loneliness at baseline was significantly associated with an increased risk of emergency presentations and higher service utilisation (more days active in SLaM and more face-to-face contacts per year) over the follow-up period (up to 7.5 years). However, loneliness was not significantly associated with crisis episodes in this subgroup.	9 stars
Schaakxs et al., 2018 [51]	Community, primary care practices and mental health care organisations	2-year course of MDD	CIDI; IDS-SR and Life Chart Interview	Age, sex, education, comorbid anxiety, antidepressant use, loneliness, social support, pain, chronic diseases, BMI, depression severity change analysis was additionally adjusted for baseline depression severity.	Logistic regression, Cox proportional hazards model, linear regression	Baseline loneliness (continuous) included as prognostic covariate in the final adjusted model examining pooled estimates for associations between age and the 2-year course of depression. For the presence of any depression diagnosis (but also for MDD and dysthymia separately), associations with age lost significance. This loss [in model overall significance] was mostly caused by the adjustment for loneliness, as it was the only significant prognostic factor in the model. Effect size estimates for loneliness were not reported.	N/A	Baseline loneliness was reported as the only significant prognostic factor in the fully adjusted model for presence of depression diagnosis at 2-year follow-up, attenuating the association between age and depression. However, effect size estimates for loneliness were not reported.	8 stars
van den Brink et al., 2018 [53]	Community, primary care practices and mental health care organisations	Depression remission; change in depression severity	CIDI; 30-item IDS-SR	Age, gender, years of education, baseline depression severity, comorbid dysthymic disorder, comorbid anxiety disorder and number of chronic diseases	Logistic regression; random coefficient analysis	Adjusted: Loneliness at T0 was associated with lower chance of remission from depression at T1 (2 years) (OR = 0.96, 95% CI [0.92–0.99], p < 0.02). When the authors tested the combined predictive performance of the social network variables and loneliness with social support from a partner and with social support from a close friend, loneliness was found to be an independent predictor of remission in both analyses, and in both, this association became non-significant after controlling for confounders. (Adjusted: OR = 0.97, 95% CI [0.92–1.03], p = 0.35; OR = 0.96, 95% CI [0.91–1.01], p = 0.09)	N/A	While loneliness impacts depression recovery, its effect may be mediated by social support (partner/close friend) and other confounders.	8 stars
van Beljouw et al., 2010 [52]	General practices (some respondents were receiving mental health support)	Depression severity	Inventory of Depressive Symptomatology (IDS-SR)	Baseline symptom severity, socio-demographic factors, comorbidity, social support	Multilevel repeated measures ANCOVA, multivariate linear regression	Adjusted: Baseline loneliness was associated with poorer depression outcomes at T1 (1 year) (β = 0.39, SE = 0.16, p < 0.05).	N/A	Baseline loneliness was independently associated with poorer depression outcomes at one year in a sample of primary care patients with current anxiety or depression.	8 stars
C. Receiving a specified clinical treatment (treatment response studies) (n = 3)									
Hassouneh et al., 2013 [38]	Community – Recruited from Centers for Independent Living, local disability service organizations, and other community settings.	Depressive symptoms	CES-D	Age, socioeconomic status, substance abuse, physical dependency, pain, fatigue	Mixed-effects models	Adjusted: The results of the multivariable model which includes eleven secondary outcomes that are singly significantly associated with the primary outcome of depressive symptoms indicate that seven variables remained significant in the model. Loneliness was found to be the strongest factor contributing to changes in the outcome of depressive symptoms over 6.5 months (14-week intervention + 3-month follow-up) with a positive association (β = 0.3827, p < 0.0001). No CI reported.	Intervention: Healing Pathways (HP) is a cross-disability peer implemented strengths-based cognitive behavioural group treatment program aiming to address depression.	Loneliness was the strongest predictor of depressive symptom changes over time while receiving the study intervention, showing a robust longitudinal association.	7 stars
Joo et al., 2025 [42]	Community-based	Depressive symptoms	PHQ-9	Model included time and mediator	Mixed effects longitudinal analyses	Loneliness was a partial mediator for the time effect on depression for PEERS and control groups separately. Change in loneliness scores explained −0.18 (95% CI [−0.32 to −0.06]) standard deviations at postintervention and −0.32 (95% CI [−0.48 to −0.19]) at 12 months for PEERS participants. For control participants, change in loneliness accounted for −0.1 (95% CI [−0.20 to 0.03]) and −0.16 (95% CI [−0.27 to −0.04]) at postintervention and 12 months, respectively.	PEERS Intervention: 8 weekly 45–60 min telephone peer-support sessions focused on goal setting, coping, social support, and service linkage.	Mediation of change over time: Reductions in loneliness mediated reductions in depression over time. The results of the mixed methods analysis of mediation showed that interventions that provide a social engagement component can work by decreasing loneliness among depressed older adults.	5 stars
McGillivray et al., 2023 [47]	Recruited from the community	Depression severity	PHQ-9	Loneliness, time, condition, and their interactions; baseline suicidal ideation (SIDAS) and depression severity (PHQ-9) implicitly adjusted via model	Linear mixed-effects models for repeated measures	Adjusted: Baseline loneliness was not associated with depression scores across time in either condition (T1 (post-intervention 6 weeks): B = 0.00, 95% CI [−0.67 to 0.66]; p = 0.99; T2 (3 months): B = 0.41, 95% CI [−0.37 to 1.18], p = 0.30).	Intervention: LifeBuoy is a self-guided smartphone intervention based on DBT and ACT to reduce suicidal ideation. It includes seven short modules ( ~ 5 min each) on distress tolerance, emotional regulation, and goal setting with interactive exercises.	Baseline loneliness scores were not found to significantly moderate the effect of the smartphone intervention on depression severity post intervention or at the 3-month post intervention follow-up in a sample with young adults with suicidal ideation.	6 stars

*T0=baseline, T1=1^st^ follow-up, T2=2^nd^ follow-up

** For consistency, we reported everywhere the final adjusted model. The unadjusted associations are only reported when there is a discrepancy in the direction or significance of the findings between adjusted and unadjusted models.

***ACT: Acceptance and Commitment Therapy; AUDIT-C: Alcohol Use Disorders Identification Test – Consumption; B: unstandardized regression coefficient; BMI: Body Mass Index; CES-D: Center for Epidemiologic Studies Depression Scale; Crisis episode: time to first inpatient admission or home treatment team; CI**:** Confidence Interval; DBT: Dialectical Behaviour Therapy; Emergency presentation: time to first contact with a catchment psychiatric liaison service in an emergency room setting; GAD-7: Generalized Anxiety Disorder-7; HR: Hazard Ratio; IDS-SR: Inventory of Depressive Symptomatology Self-Report version; IRR: Incidence Rate Ratio; Number of days active in SLaM per year: mean number of days within outpatient and/or inpatient spells of care; Number of face-to-face contacts per year: mean number of outpatient and community contacts; OR: Odds Ratio; PEERS: Peer Enhanced Depression Care; PHQ-9: Patient Health Questionnaire-9; PTSD: Post-Traumatic Stress Disorder; β: standardized regression coefficient

Depression-related outcomes included depression symptom severity, recurrence, persistence, course/remission and service utilisation. Symptom-based outcomes were assessed using validated measures such as the PHQ-9 [62], CES-D-10 [63], and CIDI [64]. Service utilisation outcomes were assessed using electronic health records or administrative data. Follow-up periods ranged from 3 months to 7.5 years.

#### Positive associations

Thirteen of the fifteen studies (87%) found that greater loneliness at baseline was associated with worse depression outcomes at follow-up in samples of individuals with depression, while two studies included mixed depression and anxiety samples [37, 38, 40–42, 44–46, 49–53]. Of these, ten studies reported worse clinical depression outcomes, including greater symptom severity, recurrence, reduced remission, chronicity, or persistence of depression at follow-up. These findings were from both observational and intervention studies. Across studies reporting adjusted positive associations, effect size estimates ranged from 0.38 to 0.61 for continuous depression outcomes (small to moderate effects), and from OR = 1.12 to 4.63 for categorical depression outcomes (small to large associations). One study [50] reported that recorded loneliness at baseline was associated with increased service utilisation over the 7.5-year follow-up period, including increased risk of emergency presentations (HR = 1.32, 95% CI [1.08–1.62], p = 0.01), days active in care per year (IRR = 1.12, 95% CI [1.11–1.13], p < 0.001), and face-to-face contacts per year (IRR = 1.41, 95% CI [1.36–1.47], p < 0.001), but no statistically significant association with crisis episodes (HR = 0.95, 95% CI [0.74–1.24], p = 0.72).

Two studies examined mediation pathways in older adults with depression [37, 42]. One identified loneliness as a mediator between low social support and MDD recurrence over 7 years [37], while another found that reductions in loneliness partially mediated reductions in depressive symptoms in a peer-support intervention [42]. These findings further support loneliness as a risk factor for poorer depression outcomes, even when not examined as a direct predictor. All studies employed adjusted models, indicating robust associations independent of confounding.

#### Negative or null associations

Two of the fifteen studies (13%) found a negative or null association between loneliness and depression outcomes. Chen et al. [36] reported that higher baseline loneliness was associated with greater improvement in depressive symptoms over one year in a sample of veterans (B = −1.55, 95% CI [−2.53 to −0.56], p < 0.01). The authors suggested this unexpected finding may reflect population-specific effects or regression to the mean. The study also found no association between loneliness and mental health visits at follow-up (B = −0.51, 95% CI [−3.40 to 2.39], p > 0.05). McGillivray et al. [47], an RCT of a digital self-guided Dialectical Behaviour Therapy (DBT)/Acceptance and Commitment Therapy (ACT) intervention for young adults with suicidal ideation, found no significant association between baseline loneliness and depression symptom outcomes either at post-treatment (B = 0.00, 95% CI [−0.67 to 0.66], p = 0.99) or at 3-month follow-up (B = 0.41, 95% CI [−0.37 to 1.18], p = 0.30). Loneliness did not moderate depression trajectories across conditions.

#### Intervention studies

Among the three intervention studies in this outcome category, findings were mixed. Hassouneh et al. [38], a strengths-based, peer-delivered Cognitive Behavioural Therapy (CBT) program for women with physical disabilities, found that baseline loneliness was the strongest predictor of change in depressive symptoms over time. Joo et al. [42] reported that reductions in loneliness partially mediated reductions in depressive symptoms over 12 months in a peer-support intervention trial, indicating that changes in loneliness explained part of the improvement in depression outcomes. In contrast, McGillivray et al. [47], as described above, found no relationship between loneliness and depression outcomes or intervention response.

Taken together, these findings suggest that the association between loneliness and subsequent depression was consistent across populations and study designs. The GRADE certainty of evidence was rated as high. According to NOS ratings, eleven studies (73%) were of high methodological quality (7–9 stars), and four studies (27%) were of moderate quality (5–6 stars).

### Meta-analysis of longitudinal associations between loneliness and depression outcomes

#### Categorical loneliness and binary depression outcomes

**Fig. 2:**
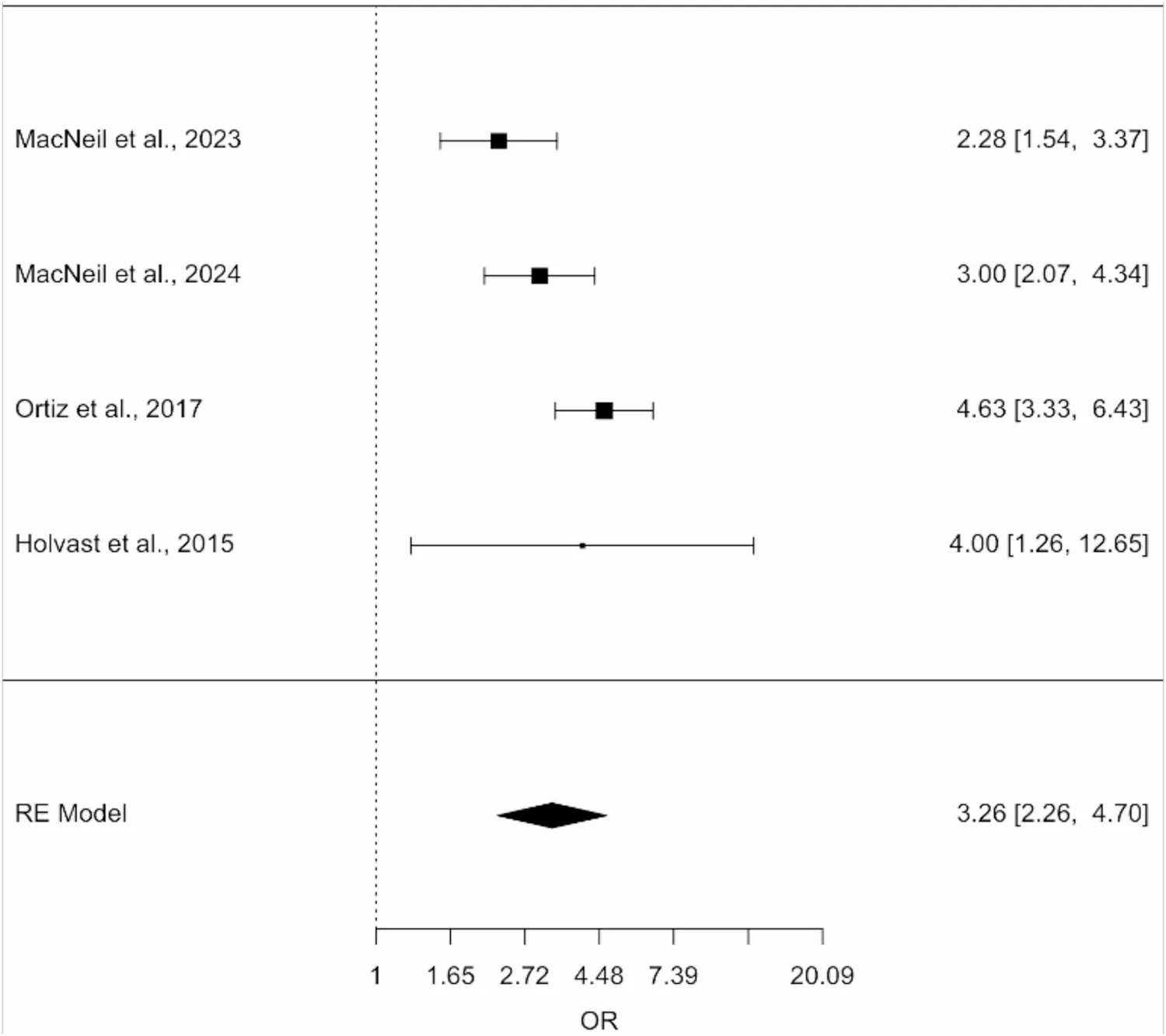
Forest plot of effect of baseline categorical loneliness on depression outcomes at follow-up

Four studies contributed adjusted estimates examining categorical loneliness (highest vs lowest category) at baseline in relation to subsequent binary depression outcomes [40, 45, 46, 49]. All studies included samples of older adults with depression (total N = 2626 participants). Two included studies were derived from the same parent cohort (CLSA), although they analysed distinct subsamples [45, 46]. Individuals who reported feeling most lonely at baseline had over three times the odds of poor depression outcomes at follow-up compared to those who reported not feeling lonely (pooled adjusted OR 3.26, 95% CI [2.26–4.70], p < 0.001; Fig. 2), with moderate between-study heterogeneity (I² = 62%). Of the four studies included in the primary analysis, only one adjusted for baseline depressive symptom severity among other sociodemographic factors [40]. The other three studies adjusted for age, sex, marital status and other sociodemographic and health-related factors but did not explicitly adjust for baseline depressive symptom severity [45, 46, 49].

#### Continuous loneliness and binary depression outcomes

**Fig. 3:**
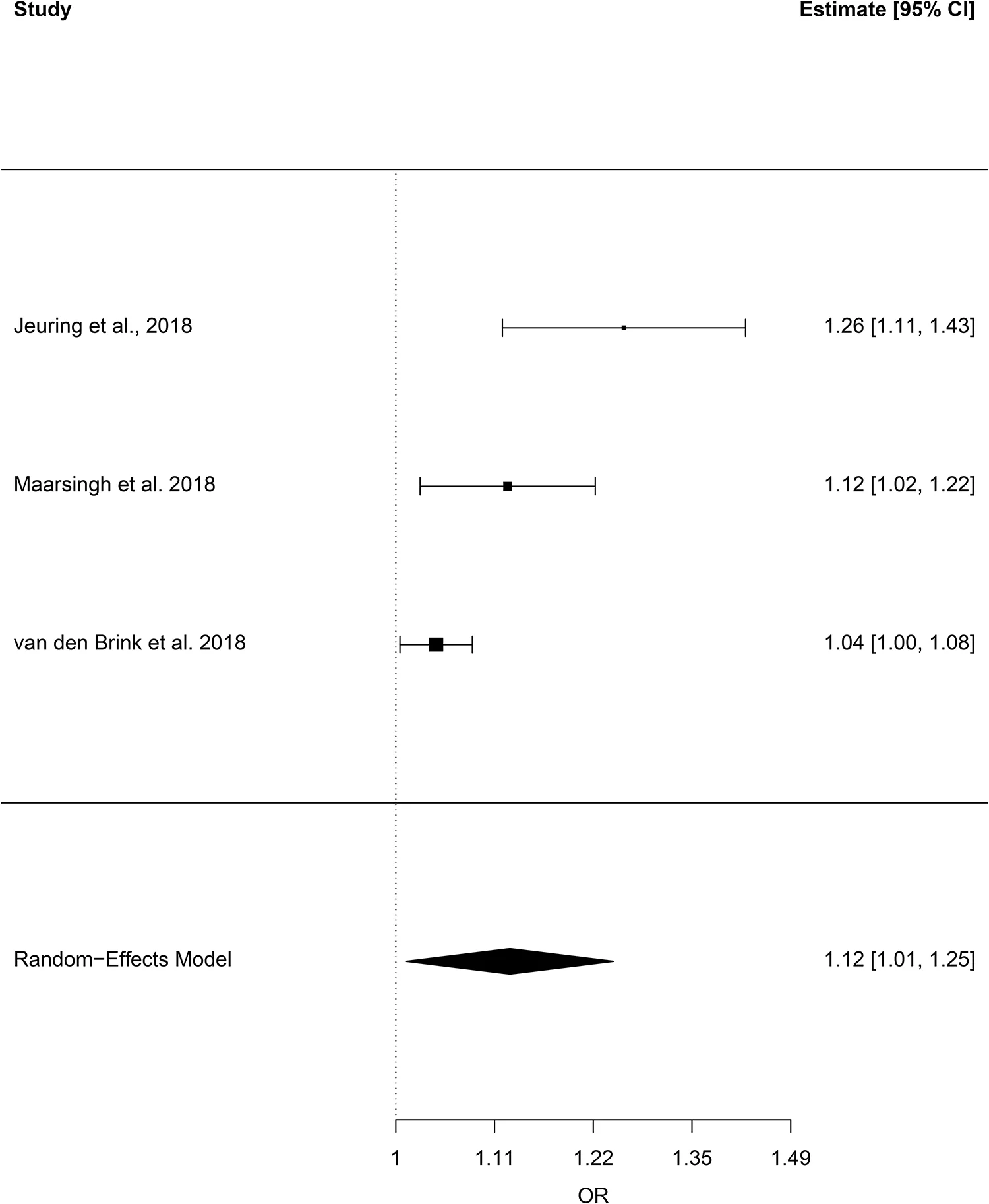
Forest plot of effect of baseline continuous loneliness on depression outcomes at follow-up (including adjusted and unadjusted estimates)

Three studies provided estimates examining continuous loneliness (per one-point increase) in relation to subsequent binary depression outcomes among individuals with MDD [41, 44, 53]. Two studies reported adjusted estimates [44, 53], whereas one study reported an unadjusted estimate [41]. Both studies with adjusted estimates adjusted for baseline symptom severity. Together, all three studies included 2026 participants, comprising both older adult and mixed adult samples aged 18–90 years. In the meta-analysis including all three studies, each one-point increase in baseline loneliness was associated with a 12% increase in the odds of poor depression outcomes at follow-up (pooled OR = 1.12, 95% CI [1.01–1.25], p = 0.031; see Table 3), although between-study heterogeneity was substantial (I² = 81%). The inclusion of an unadjusted estimate and the substantial heterogeneity reduce confidence in this pooled estimate. Therefore, these findings should be interpreted cautiously Fig. 3.

**Table 3:** Pooled ORs for associations between baseline loneliness and poor depression outcomes at follow-up

Loneliness Type	K	Type of effect estimates	Pooled OR (95% CI)	p	I²
Categorical	4	Adjusted	3.26 (2.26–4.70)	<0.001	62%
Continuous	3	Adjusted + unadjusted	1.12 (1.01–1.25)	0.031	81%
Combined (exploratory)	6	Adjusted	2.16 (1.28–3.66)	0.004	99%

#### Exploratory analysis: Combined categorical and continuous loneliness measures

An exploratory random-effects meta-analysis combining categorical and continuous loneliness measures across six studies yielded a pooled adjusted OR of 2.16 (95% CI [1.28–3.66], p = 0.004), with considerable between-study heterogeneity (I² = 99%). We also conducted an additional moderator analysis with measurement type (categorical versus continuous loneliness) as the moderator, which showed that measurement type accounted for approximately 92% of the between-study heterogeneity. These findings suggest that the analyses estimate different underlying effect parameters. We therefore retained the stratified analyses as the primary analyses because they provide more interpretable pooled effect estimates. The exploratory combined analysis is presented in Additional file: Appendix 7.

#### Loneliness and suicidal ideation

Six studies examined the relationship between baseline loneliness and suicidal ideation at follow-up (see Table 4) [34–36, 43, 47, 55]. Suicidal ideation was assessed using a range of measures, including validated suicide-specific instruments (e.g., the Suicidal Ideation Attributes Scale [SIDAS] and the Beck Scale for Suicide Ideation), suicidality items from validated depression scales (e.g., the Beck Depression Inventory-II [BDI-II] and the Inventory of Depressive Symptomatology [IDS]), item 9 of the PHQ-9 (which does not distinguish suicidal ideation from non-suicidal self-harm ideation), and, in one study, a single self-report question [34]. Two studies were conducted in general population samples with elevated levels of anxiety and depression (met screening thresholds) [34, 35], three involved clinical samples of individuals with depression using mental health services [36, 43, 55], and one examined a sample of young adults with suicidal ideation and elevated depressive symptoms (Mean PHQ-9 score = 17.15 (SD 5.64)) receiving a specific clinical treatment [47]. Follow-up periods ranged from one month to nine years.

**Table 4:** Study findings for suicidal ideation outcomes (N = 6)

Author(s), Year	Setting	Main CMD outcome(s)	Main CMD outcome measure(s)	Adjusted for	Statistical methods	Results of the longitudinal association of baseline loneliness with CMD outcomes at follow-up (Effect size, 95% CI, P-value)	Intervention (if applicable)	Conclusions	NOS score
A. General Population (care unknown) (n = 2)									
Antonelli-Salgado et al., 2021 [34]	Community – online survey	Suicidal ideation	Self-report to a single question	Age, gender, sexual orientation, marital status, income, geographical area, education, profession, unemployment, financial crisis during the pandemic, quality of family relationships, quality of friend relationships, religion, meditation, sleep quality, physical activity, childhood trauma, previous suicide attempt, family history of suicide, GAD-7, PHQ-9, benzodiazepines use and AUDIT-C	Multinomial logistic regression analysis	Adjusted: Baseline loneliness was directly associated with the incidence of suicidal ideation between T0 and T1 (1 month) (OR = 2.12, 95% CI [1.06–4.24], p = 0.033).	N/A	Loneliness was consistently associated with the incidence of suicidal ideation in a one-month follow-up during the initial stage of the COVID-19 pandemic in a general population sample with anxiety symptoms.	6 stars
Brown et al., 2023 [35]	Community – recruited via social media and online platforms	Suicidal ideation	BDI-II Suicide Item	Baseline suicidal ideation and social anxiety	Mediation Analysis with bootstrapping	Adjusted: Loneliness at T1 (14-day daily diary period) did not predict suicidal ideation at T2 (1.5 months) when controlling for baseline social anxiety and baseline suicidal ideation (β = 0.08, p = 0.25). Loneliness did not mediate the social anxiety-suicidal ideation relationship (indirect effect: β = 0.03, Bootstrap CIs [−0.02 to 0.08]).	N/A	Loneliness did not significantly mediate the relationship between social anxiety and suicidal ideation at 1.5-month follow-up in a sample of community adults with elevated depression and/or social anxiety.	5 stars
B. Using mental health services (n = 3)									
Chen et al., 2020 [36]	Primary care and other services at a Veterans Health Administration (VHA) facility	Change in suicidal ideation	PHQ-9 (item 9)	Age, gender, race/ethnicity, AUDIT-C screening score, PTSD screening score, medical comorbidity score, financial hardship, other social connectedness variables	Multivariable regression analysis	Adjusted: Higher baseline loneliness was associated with decreased suicidal ideation from T0 to T1 (1 year) (B = −0.21, 95% CI [not reported], p < 0.05).	N/A	Unexpectedly, higher levels of baseline loneliness were associated with greater improvement in suicidal ideation over one year in a sample of veterans with depression. The authors suggest that these results contrast with previous studies and may be due to population-specific characteristics and regression to the mean.	7 stars
Kivelä et al., 2019 [43]	Clinical sample – recruited from community and health care settings. Some were on medication.	Course and persistence of suicidal ideation	Beck Scale for Suicide Ideation (BSSI)	Sociodemographic (age, gender, marital status, education, employment), clinical factors (depression, anxiety, comorbidity, symptom severity, medication use, suicide attempt history), baseline predictors (insomnia symptoms, hopelessness, loneliness, BPD traits, childhood trauma, negative life events)	Linear mixed-effects growth models and logistic regression	Growth model (N = 230) Loneliness tested in preliminary models and did not reach significance (p > 0.05). Therefore, it was excluded from final model. Persistence model (N = 195) Adjusted: Loneliness at T0 (mean of baseline, 2-year, and 4-year assessments) was not associated with persistent suicidal ideation at T1 (present at both 6-year and 9-year follow-up) (OR = 1.03, 95% CI [0.88–1.20], p > 0.05).	N/A	Loneliness was not longitudinally associated with the course or persistence of suicidal ideation in a sample of individuals with CMDs and suicidal ideation.	7 stars
Zuidersma et al., 2025 [55]	Clinical sample – Mental healthcare facilities and general practitioners	Suicidal ideation trajectory over 6 years	IDS item on suicidal ideation	Each model included only the determinant under investigation and total IDS-score (without the item on suicidal ideation) at baseline.	Multinomial regression analysis	Loneliness at baseline was associated with worse suicidal ideation trajectories independent of baseline depression severity. Adjusted ORs per point increase in loneliness (reference = class 5 “no thoughts”): Class 1 (severe transient): OR = 1.14, 95% CI [1.02–1.28]* Class 2 (severe persistent): OR = 1.15, 95% CI [1.01–1.32] Class 3 (mild increasing): OR = 1.14; 95% CI [1.03–1.26] * Class 4 (moderate persistent): OR = 1.23; 95% CI [1.12–1.34]* *All p < 0.05 (class 4 p < 0.001) Higher baseline loneliness increased odds of belonging to any trajectory with suicidal ideation.	N/A	Loneliness was consistently and independently of depressive symptom severity higher in all four classes with at least some suicidal ideation or thoughts of meaning of life, suggesting it to be an important risk factor for suicidal ideation or loss of meaning of life.	6 stars
C. Receiving a specified clinical treatment (treatment response studies) (n = 1)									
McGillivray et al., 2023 [47]	Recruited from the community	Suicidal ideation	Suicidal Ideation Attributes Scale (SIDAS)	Loneliness, time, condition, and their interactions; baseline suicidal ideation (SIDAS) and depression severity (PHQ-9) implicitly adjusted via model	Linear mixed-effects models for repeated measures	Adjusted: Baseline loneliness was not associated with suicidal ideation scores across time in either condition (B = 1.10, 95% CI [−0.25 to 2.46], p = 0.11) at T1 (post intervention 6 weeks); B = 0.43, 95% CI [−1.25 to 2.12]; p = 0.61 at T2 (3 months post-intervention follow-up).	Intervention: LifeBuoy is a self-guided smartphone intervention based on DBT and ACT to reduce suicidal ideation. It includes seven short modules ( ~ 5 min each) on distress tolerance, emotional regulation, and goal setting with interactive exercises.	Baseline loneliness scores were not found to significantly moderate the effect of the smartphone intervention on suicidal ideation post intervention or at the 3-month post intervention follow-up in a sample of young adults with suicidal ideation.	6 stars

*T0=baseline, T1=1^st^ follow-up, T2=2^nd^ follow-up

** For consistency, we reported everywhere the final adjusted model. The unadjusted associations are only reported when there is a discrepancy in the direction or significance of the findings between adjusted and unadjusted models.

***ACT: Acceptance and Commitment Therapy; AUDIT-C: Alcohol Use Disorders Identification Test – Consumption; B: unstandardized regression coefficient; BDI-II: Beck Depression Inventory–II; BPD: Borderline Personality Disorder; BSSI: Beck Scale for Suicide Ideation; CI: Confidence Intervals; CMD: Common Mental Disorder; DBT: Dialectical Behaviour Therapy; GAD-7: Generalized Anxiety Disorder-7; IDS: Inventory of Depressive Symptomatology; N/A: Not applicable; NOS: Newcastle–Ottawa Scale; OR: Odds Ratio; PHQ-9: Patient Health Questionnaire-9; PTSD: Post-Traumatic Stress Disorder; SIDAS: Suicidal Ideation Attributes Scale; VHA: Veterans Health Administration; β: standardized regression coefficient.

##### Positive associations

Two of the six studies found a positive association. Antonelli-Salgado et al. [34] found that greater loneliness at baseline was associated with higher incidence of suicidal ideation in a one-month follow-up during the COVID-19 pandemic in a general population sample with anxiety symptoms (OR = 2.12, 95% CI [1.06–4.24], p = 0.033). Similarly, Zuidersma et al. [55] found that loneliness at baseline was associated with worse suicidal ideation trajectories over six years, independent of baseline depression severity, in a clinical sample of older adults with diagnosed depression drawn from the NESDO cohort (ORs ranging from 1.14 to 1.23 per-point increase in loneliness; all p < 0.05).

##### Negative or null associations

Three studies reported no significant association between loneliness and subsequent suicidal ideation. Kivelä et al. [43], in a long-term (up to 9-year) clinical cohort of individuals with CMDs and suicidal ideation, found that loneliness was not associated with either the trajectory or persistence of suicidal ideation (adjusted OR = 1.03, 95% CI [0.88–1.20], p > 0.05). McGillivray et al. [47], in a digital intervention study of young adults with suicidal ideation and elevated depressive symptoms (Mean PHQ-9 score = 17.15 (SD 5.64)), found no significant effect of baseline loneliness on suicidal ideation outcomes, either at post-treatment (B = 1.10, 95% CI [−0.25 to 2.46], p = 0.11) or at three-month follow-up (B = 0.43, 95% CI [−1.25 to 2.12], p = 0.61). These findings mirrored those reported in the previous section on depression outcomes, where loneliness was also not found to significantly predict depression severity in this trial. Brown et al. [35], using a daily diary design in adults with elevated depression and/or social anxiety symptoms, found that loneliness did not significantly mediate the relationship between social anxiety and suicidal ideation at 1.5-month follow-up.

Finally, Chen et al. [36] reported an inverse relationship: higher loneliness levels at baseline were associated with greater improvement in suicidal ideation, assessed using PHQ-9 item 9, over one year in a sample of veterans with depression (B = −0.21; p < 0.05). The authors suggested that this unexpected result may reflect regression to the mean or population-specific factors.

Taken together, the current evidence is mixed and does not suggest a consistent association between loneliness and subsequent suicidal ideation among people with CMDs. The GRADE certainty of evidence was low, reflecting serious concerns about inconsistency in findings and imprecision due to small sample sizes. Four studies were of moderate methodological quality (5–6 stars) and two were of high quality (7 stars).

#### Loneliness and anxiety or mixed CMD outcomes

Five studies examined the relationship between baseline loneliness and anxiety or mixed CMD outcomes at follow-up (see Table 5) [39, 48, 50, 52, 54]. Three studies focused specifically on anxiety [50, 52, 54], while two investigated the severity of mixed CMD outcomes [39, 48]. Outcomes included anxiety severity, service utilisation and CMD severity.

**Table 5:** Study findings on anxiety and mixed CMD outcomes (N = 5)

Author(s), Year	Setting	Main CMD outcome(s)	Main CMD outcome measure(s)	Adjusted for	Statistical methods	Results of the longitudinal association of baseline loneliness with CMD outcomes at follow-up (Effect size, 95% CI, P-value)	Intervention (if applicable)	Conclusions	NOS score
Mixed outcomes (n = 2)									
Hirshberg et al., 2025 [39]	Treatment response study	Psychological distress	NIH Perceived Stress Scale (10-item) + PROMIS Anxiety & Depression scales (adaptive); z-scored and aggregated	Baseline distress score	Latent growth structural equation mediation model (LGSEM)	Assignment to the HMP predicted a 0.31 SD pre- to post-test reduction in loneliness (p < 0.001) which significantly mediated 62.5% of the HMP’s total effect on follow-up distress (b = −0.10, p < 0.001). The direct effect of HMP assignment on follow-up distress was not significant in the loneliness model (b = −0.05, p = 0.148). In the exploratory multiple mediator model, the four mediators combined explained nearly 62% of the total direct effect of assignment to the HMP on follow-up distress reductions. The majority of this effect was attributable to intervention-related reductions in loneliness (61.7% of the indirect effect).	Healthy Minds Program, HMP: 4-week smartphone-based meditation intervention based on the Awareness, Connection, Insight, and Purpose (ACIP) model of well-being.	The intervention significantly reduced loneliness, and reductions in loneliness significantly mediated reductions in psychological distress at 3-month follow-up. Loneliness accounted for the largest proportion of the indirect effect in mediation models, suggesting it may be a key psychological pathway through which the intervention reduced distress.	6 stars
Nuyen et al., 2020 [48]	Community – General population (care unknown)	Severity of CMD (mild/moderate vs severe)	CIDI	Gender, age, education, living situation, job status, and household income situation, negative life events, chronic physical disorders, BMI, perceived social support	Multivariate multinomial logistic regression	In the subgroup with CMD at baseline: Adjusted: For respondents with CMD at baseline, loneliness at T0 was not associated with persistent mild–moderate CMD at T1 (3 years) (Relative Risk Ratio [RRR] = 1.15, 95% CI [0.54–2.47]). Unadjusted: For respondents with CMD at baseline, loneliness at T0 significantly predicted persistent severe CMD at follow-up (Relative Risk Ratio [RRR] = 2.48, 95% CI [1.21–5.11], p < 0.05). Adjusted: The association became non-significant after adjusting for perceived social support (Adjusted RRR = 1.80, 95% CI [0.83–3.91]).	N/A	There was no significant relationship between loneliness and persistent mild–moderate CMD at follow-up across all models. Loneliness predicted persistent severe CMD in unadjusted models, but social support attenuated this effect in adjusted models.	9 stars
Anxiety outcomes (n = 3)									
van Beljouw et al., 2010 [52]	General practices (some respondents were receiving mental health support) – Using mental health services	Anxiety symptom severity	Beck Anxiety Inventory (BAI)	Baseline symptom severity, socio-demographic factors, comorbidity, social support	Multilevel repeated measures ANCOVA, multivariate linear regression	Unadjusted: Baseline loneliness was significantly associated with poor anxiety outcomes at T1 (1 year) (β = 0.40, SE = 0.12, p < 0.05). Adjusted: Baseline loneliness was not significantly associated with poor anxiety outcomes at T1 (1 year) after adjustments (β = −0.02, SE = 0.10).	**N/A**	After adjustment, baseline loneliness was not associated with anxiety outcomes at 1 year follow-up.	8 stars
Parmar et al., 2024 [50]	SLaM Mental Healthcare Provider – using mental health services	Crisis episode*** Emergency presentation*** Number of days active in SLaM per year*** Number of face-to-face contacts per year***	Electronic health record data	Number of documents available to review, sociodemographic variables, Psychotropic medications, Psychiatric diagnoses, Physical health conditions and medications for physical health	Cox proportional hazard models; Poisson regression analyses	Adjusted Models: Crisis episode**:** Recorded loneliness at baseline in patients with anxiety was not significantly associated with an increased risk of crisis episodes at T1 (7.5 years follow-up) (HR = 1.11, 95% CI [0.65–1.90], p = 0.70). Emergency presentation**:** Recorded loneliness at baseline in patients with anxiety was not significantly associated with higher risk of emergency presentations at T1 (7.5 years follow-up) (HR = 0.92, 95% CI [0.62–1.37], p = 0.68). Number of days active in SLaM per year: Recorded loneliness at baseline in patients with anxiety was not significantly associated with the number of days active in SLaM per year at T1 (7.5 years follow-up) (IRR = 0.95, 95% CI [0.88–1.02], p = 0.18). Number of face-to-face contacts per year**:** Recorded loneliness at baseline in patients with anxiety was significantly associated with an increased number of face-to-face contacts per year at T1 (7.5 years follow-up) (IRR = 1.24, 95% CI [1.13–1.37], p < 0.001).		Baseline loneliness in patients with anxiety was significantly associated only with increased face-to-face contacts per year over 7.5 years. No significant associations were found with crisis episodes, emergency presentations, or days active in SLaM. Loneliness may drive increased clinician engagement in anxiety patients, warranting further research into targeted interventions.	9 stars
Zech et al., 2025 [54]	Treatment response study	Social anxiety	Social Phobia Inventory (SPIN)	Baseline social anxiety	Post-hoc exploratory analyses; Multiple linear regression (SEM framework using lavaan, maximum likelihood)	Greater baseline loneliness was associated with higher post-treatment social anxiety (β = 0.775, p = 0.012, sr² = 0.052) as well as higher follow-up social anxiety (β = 0.990, p = 0.004, sr² = 0.088). Among those in the UB condition, however, baseline loneliness also did not predict social anxiety at either posttreatment (β = 0.621, p = 0.099, sr² = 0.029) or follow-up (β = 0.345, p = 0.269, sr² = 0.010)	Safety Behaviour (SB) fading condition: a 28-day digital safety behaviour treatment for social anxiety, consisting of brief, daily checklists delivered to participants’ mobile devices.	Higher baseline (pre-treatment) loneliness was associated with poorer social anxiety outcomes at follow-up in the safety behaviour fading condition, suggesting loneliness may limit responsiveness to this intervention.	5 stars

*T0=baseline, T1=1^st^ follow-up, T2=2^nd^ follow-up

** For consistency, we reported everywhere the final adjusted model. The unadjusted associations are only reported when there is a discrepancy in the direction or significance of the findings between adjusted and unadjusted models.

*** ACIP: Awareness, Connection, Insight, and Purpose; ANCOVA: Analysis of Covariance; BAI: Beck Anxiety Inventory; BMI: Body Mass Index; CI: Confidence Interval; CIDI: Composite International Diagnostic Interview; CMD: Common Mental Disorder; Crisis episode: Time to first inpatient admission or home treatment team; Emergency presentation: Time to first contact with a catchment psychiatric liaison service in an emergency room setting; HMP: Healthy Minds Program; HR: Hazard Ratio; IRR: Incidence Rate Ratio; LGSEM: Latent Growth Structural Equation Mediation Model; N/A: Not Applicable; NIH: National Institutes of Health; NOS: Newcastle–Ottawa Scale; Number of days active in SLaM per year: Mean number of days within outpatient and/or inpatient spells of care; Number of face-to-face contacts per year: Mean number of outpatient and community contacts; PROMIS: Patient-Reported Outcomes Measurement Information System; RRR: Relative Risk Ratio; SB: Safety Behaviour; SD: Standard Deviation; SE: Standard Error; SEM: Structural Equation Modelling; SLaM: South London and Maudsley; SPIN: Social Phobia Inventory; sr²: Squared Semi-Partial Correlation; UB: Unhealthy Behaviour; b: Unstandardized Regression Coefficient; β: Standardized Regression Coefficient

Findings related to anxiety were limited and inconsistent. van Beljouw et al. [52] found no significant association between baseline loneliness and anxiety symptoms at one-year follow-up after adjustment (β = −0.02, SE = 0.10). Parmar et al. [50] reported mixed effects: loneliness was not associated with crisis episodes, emergency presentations, or days active in care, but was significantly associated with increased face-to-face contacts per year (IRR = 1.24, 95% CI [1.13–1.37], p < 0.001). Zech et al. [54] found that higher baseline loneliness was associated with higher post-treatment and one-month follow-up social anxiety after adjustment for baseline symptoms (β = 0.775, p = 0.012; β = 0.990, p = 0.004). This effect was observed only in the intervention group and not in the control condition, suggesting loneliness may limit responsiveness to this intervention.

In one of the two studies on mixed CMD outcomes, Nuyen et al. [48] examined mixed CMD severity in a general population sample of individuals with CMD at baseline. Loneliness at baseline did not significantly predict persistent mild-to-moderate CMD at three-year follow-up (Adjusted RRR = 1.15, 95% CI [0.54–2.47]), nor persistent severe CMD after controlling for perceived social support (Adjusted RRR = 1.80, 95% CI [0.83–3.91]). Although a significant unadjusted association was initially observed (RRR = 2.48, 95% CI [1.21–5.11]), this was attenuated in adjusted models. Hirshberg et al. [39], analysing data from a randomised controlled trial of a smartphone-based well-being intervention, reported that assignment to the intervention significantly reduced loneliness, and that reductions in loneliness mediated reductions in psychological distress at 3-month follow-up (indirect effect b = −0.10; p < 0.001). The direct effect of intervention assignment on distress was not significant when loneliness was included in the model.

Three studies were of high methodological quality (8–9 stars) and two were of moderate quality (5–6 stars). However, the GRADE certainty of evidence for anxiety was rated as low, and that for mixed CMD outcomes was rated as very low. This was due to serious concerns about inconsistency, imprecision (e.g., small number of studies and conflicting results), and potential publication bias.

## Discussion

### Summary of findings

This systematic review examined the longitudinal associations between baseline loneliness and follow-up outcomes in adults with CMDs. This review addressed an important gap in the literature regarding the prognostic role of loneliness in those already affected by CMDs, irrespective of whether they were receiving treatment for their CMD. Twenty-two studies were included, with the majority (68%) focusing on depression-related outcomes. Evidence in relation to suicidal ideation, anxiety, and mixed CMD outcomes was limited and inconsistent.

We found high-quality, consistent evidence that greater baseline loneliness is associated with subsequent poorer depression outcomes (more severe depressive symptoms, lower remission rates, increased recurrence, persistence of depression and increased service use) among adults with CMDs affected by depressive disorders. This association was observed across clinical samples, community cohorts, and intervention trials, suggesting prognostic relevance in diverse contexts. Most studies were rated to be of high methodological quality, as they employed validated measures and adjusted for key confounders. While two studies reported null or negative findings, these appeared to reflect population-specific factors or intervention-related differences, rather than a contradiction of the overall pattern of effects.

Our meta-analysis of four studies (all in older adults with depression) showed that individuals reporting loneliness at baseline had over three times the odds of poor depression outcomes at follow-up compared with those not lonely (pooled adjusted OR = 3.26, 95% CI [2.26–4.70], p < 0.001). A separate meta-analysis of continuous loneliness measures (three studies) suggested a 12% per-point increase in the odds of poor depression outcomes (pooled OR = 1.12, 95% CI [1.01–1.25], p = 0.031). However, this estimate included one unadjusted study, substantial between-study heterogeneity (I² = 81%), and should therefore be interpreted cautiously.

An important consideration is that only one of the four studies in the binary loneliness meta-analysis adjusted for baseline depression severity, raising the possibility that loneliness partly reflects greater initial severity. However, the broader narrative synthesis—which included multiple studies adjusting for baseline severity—showed consistent associations with poorer outcomes suggesting independent effects beyond initial severity. Findings should be interpreted with caution, and no causal inferences can be made.

In contrast, the evidence linking loneliness to suicidal ideation in adults with CMDs was mixed and of low certainty drawn predominantly from moderate quality studies. While two well-adjusted longitudinal cohort studies found that higher loneliness was associated with subsequent suicidal ideation, three studies reported null findings and one study an inverse association. Positive associations were observed in both short-term (1 month) and long-term (6 years) follow-ups, but inconsistencies across populations, settings, and analytic approaches suggest substantial heterogeneity in this relationship.

Findings for anxiety outcomes were limited and mixed. One study reported no association between baseline loneliness and subsequent anxiety symptoms after adjustment in a sample with mixed depression and anxiety, while another found mixed effects for service utilisation in people with anxiety, with positive associations between baseline loneliness and face-to-face outpatient and community contacts at follow-up. A third study found that higher baseline loneliness was associated with poorer social anxiety outcomes at follow-up within the intervention arm only in a sample of people with social anxiety. Evidence for mixed CMD outcomes was limited and inconsistent, with one population-based study reporting no significant adjusted associations and one intervention study indicating that reductions in loneliness were associated with reductions in follow-up psychological distress. Overall, certainty of evidence for anxiety was low and for mixed CMD outcomes very low.

With respect to treatment outcomes, five studies evaluated whether loneliness affected intervention response. Three trials examined baseline loneliness as a prognostic factor for symptom trajectories: higher loneliness was associated with subsequent poorer depression outcomes in one CBT programme and poorer social anxiety outcomes in one digital intervention arm but showed no association with changes in depressive symptoms or suicidal ideation in another digital trial. Two additional studies found that reductions in loneliness mediated improvements in depressive symptoms or psychological distress over time. Further research is needed to clarify whether and how loneliness influences treatment response in CMDs.

Overall, these findings suggest that loneliness is associated with poor subsequent depression outcomes, an association that was consistent across the narrative synthesis and all meta-analyses. However, further work is needed to understand its role across CMDs and within treatment settings.

### Putting findings into context

Our findings align with theoretical models and systematic reviews that position loneliness as both a precursor and a perpetuating factor in the development and maintenance of depression [16, 17, 23]. The last review on this topic by Wang et al. [23] reviewed 34 longitudinal studies and found evidence that poor perceived social support and greater loneliness were associated with poorer depression outcomes. However, only two studies in that review assessed loneliness specifically. Our review expands these findings by providing a more focused synthesis, specifically on loneliness. It includes a larger number of relevant depression studies with longer follow-up durations, offering greater certainty about the longitudinal associations with meta-analytic pooling quantifying the strength of the association. A related review by Mann et al. [17] synthesised evidence from longitudinal studies investigating the relationship between loneliness and the onset of mental health problems in the general population, finding that loneliness at baseline is associated with the subsequent onset of depression. Our findings complement these by focusing on individuals already affected by CMDs, highlighting the role of loneliness in the persistence and severity of depression outcomes. Notably, our review found some evidence that loneliness may contribute to increased demand on mental health services for people with depression, suggesting that lonely individuals may continue to struggle despite engagement with care. Although preliminary, these findings raise important questions about the adequacy of standard treatments in addressing the needs of people with CMDs who are also lonely.

The consistent association between loneliness and subsequent poorer depression outcomes may reflect several underlying mechanisms. Loneliness is associated with increased rumination, negative social cognition, and reduced engagement in rewarding activities [65]. Ruminative thinking about being lonely has been found to be a mediator between loneliness and depressive symptoms [66]. Negative social cognition has also been proposed as a way through which loneliness might contribute to depressive symptoms via fostering feelings of rejection and social withdrawal [2, 67]. Moreover, chronic loneliness has been linked to dysregulated stress responses, inflammation, and altered neural functioning, which may biologically predispose individuals to more severe or persistent depressive symptoms [68]. Recent evidence from a large Mendelian randomisation study found that, unlike many physical conditions, where observational associations with loneliness were not supported genetically, depression stood out as a condition where both lines of evidence align. This suggests that loneliness is not just a correlate but may be a causal contributor to depression [69]. In addition to these intra-personal processes, loneliness may reflect a lack of supportive others to encourage help-seeking or re-engagement with social and occupational roles. The absence of social companions may also reduce opportunities for meaningful activity, thereby undermining informal behavioural activation—an evidence-based mechanism for depression recovery [70]. These findings underscore the importance of identifying and addressing loneliness in individuals with depression. Interventions that target both the social and cognitive features of loneliness may be critical to improving long-term outcomes and should be prioritised in future research.

Our review did not find consistent evidence for a prospective association between loneliness and suicidal ideation in individuals with CMDs. This is consistent with the findings of the McClelland et al. [12] review within clinical populations, where evidence from psychiatric inpatient, veteran, and depression samples was mixed. Although the overall findings of the McClelland review [12] concluded that loneliness prospectively predicted suicidal ideation and suicidal behaviour, this overall association was largely driven by studies conducted in general population samples. McClelland et al. [12] adopted a transdiagnostic framework and included heterogeneous populations, whereas our review focused specifically on longitudinal studies within CMD samples. Furthermore, the studies contributing suicidal ideation outcomes in our review were drawn from mixed CMD populations (e.g., depression and anxiety), which may have further increased clinical heterogeneity. Another possible explanation for these mixed findings is the considerable heterogeneity in how suicidal ideation was defined and measured across studies, both in our review and in the McClelland review. Hughes and Pitman (2026) [71] highlighted that inconsistent terminology and definitions across suicide research hinder comparability between studies. It is also possible that loneliness influences suicidal ideation indirectly, through mediators such as hopelessness or depression, as proposed by the interpersonal theory of suicide [72, 73], and subsequent meta-analytic evidence demonstrating robust associations between thwarted belongingness, perceived burdensomeness, and suicidal ideation [74].

Furthermore, a recent systematic review by Lu et al. [75] identified a moderate association between loneliness and suicidality in healthy adults and individuals with depressive disorders, based primarily on cross-sectional studies. However, our findings suggest that this relationship may not reliably extend to longitudinal associations in CMD populations. Unlike our review, which focused exclusively on longitudinal studies in clinical CMD samples, these broader reviews included mixed populations and—for Lu et al. [75]—cross-sectional designs. Our results should be interpreted with caution, as the GRADE certainty of evidence was low due to the limited number of longitudinal studies, inconsistent findings, and small sample sizes. These differences highlight the importance of standardised methodologies and longer-term studies to clarify the prospective role of loneliness in suicidal ideation among people with CMDs.

The mixed findings for anxiety outcomes in our review are consistent with the patterns observed in the broader literature, where weaker or less consistent associations between loneliness and anxiety are noted compared with depression [23, 76, 77]. A cross-sectional study by Beutel et al. [76] found that loneliness was more strongly associated with depression than anxiety in general populations, potentially due to the distinct cognitive and social mechanisms underlying these disorders. The null finding reported by one study in our review, after adjustment for confounders, suggests that loneliness may interact with other factors, such as baseline anxiety severity or co-occurring depression, complicating its prognostic role.

### Strengths and limitations

Key strengths of this review include a comprehensive search strategy, the inclusion of diverse CMD populations and settings, and detailed independent quality and certainty assessments using the NOS and GRADE criteria. We also screened and included studies published in languages other than English, reducing language bias and increasing the comprehensiveness of the review. Despite these strengths, there are a few limitations that should be acknowledged. First, despite following standard best-practice approaches in our search strategy—including metadata-focused searching with free-text terms combined with subject headings (Cochrane Handbook for Systematic Reviews of Interventions, Chapter 4) [78]—the database searches may have had lower sensitivity to studies where loneliness was assessed as one of multiple psychosocial risk factors but not explicitly mentioned in indexed fields. Supplementary strategies, such as citation checking of relevant reviews and grey literature searching, were used to help mitigate this risk. Second, few studies distinguished between types of loneliness (e.g., emotional and social), meaning we could not examine whether different dimensions of loneliness are differentially associated with outcomes.

Moreover, in observational studies, the baseline often represents an arbitrary point in an individual’s illness trajectory. This makes it difficult to determine temporal precedence with certainty, particularly given the known bidirectionality between loneliness and CMD symptoms. Nevertheless, longitudinal designs remain superior to cross-sectional studies in terms of causal inference, even if they do not capture full histories. Furthermore, heterogeneity in treatments received by participants, especially in non-intervention studies, may have confounded associations. In intervention studies, pooling experimental and control groups to assess the influence of loneliness may have diluted effects, as control groups often received no active treatment, potentially weakening observed associations.

Although our review had no language restrictions and the included studies were conducted across the globe, most of the evidence was concentrated in high-income Western countries (91%), particularly the Netherlands (41%) and the USA (27%). Only two studies were conducted in Latin America (Brazil and Mexico), and none were conducted in Africa, Asia, or the Middle East. Though the findings were consistent, with no clear geographical differences identified, the geographical concentration of the evidence may still limit the generalisability of findings across different cultural and socioeconomic contexts, where the experience and meaning of loneliness may differ.

Finally, nine of the included studies—many of those contributing to the evidence on depression—focused on older adult populations. This is particularly relevant to the meta-analysis of categorical loneliness, where all four contributing studies were in older adults, potentially limiting generalisability of the pooled ORs to younger or more diverse populations with CMDs. In addition, our review focused predominantly on adult samples and did not include studies exclusively examining children or adolescents. Given that social relationships and developmental factors play a critical role in the onset and course of CMDs during adolescence, future research should examine whether loneliness has similar or distinct prognostic implications in younger populations.

### Implications for research

This systematic review highlights an important research gap: the limited longitudinal evidence for the role of loneliness in CMD outcomes beyond depression. Understanding whether loneliness worsens anxiety or other mental health outcomes over time is crucial for determining which clinical groups may benefit most from loneliness-targeted interventions. Our review also indicates that research examining loneliness in depression has predominantly focused on older adults, reflecting a broader trend within the loneliness literature. Further longitudinal research is needed to investigate the impact of loneliness across younger and mid-life adult populations. Further research examining the longitudinal relationship between loneliness and CMD outcomes is also needed in regions outside Europe and the USA.

Future research should also investigate how loneliness interacts with psychological therapies for CMDs. Most studies reviewed here focused on naturalistic outcomes, and very few examined whether loneliness moderates or mediates treatment response. Given the established links between loneliness and depression, we need to know how best to support people with depression who are also lonely. If loneliness is consistently associated with poorer treatment response, particularly in depression, this would support the development of bespoke interventions for people who are both lonely and depressed. Promising programmes, such as Community Navigators—a novel co-produced social intervention aiming to reduce loneliness in people with treatment-resistant depression—show early potential and require further testing in full-scale RCTs [79, 80].

### Implications for practice

Despite the current paucity of robust, evidence-based interventions specifically targeting loneliness in clinical populations, mental health professionals should not overlook its importance in clinical formulation and treatment planning.

The consistent association between loneliness and depression—both as a risk factor for its onset and as a prognostic factor for poorer outcomes—highlights the need for further research into the potential benefits of routinely assessing loneliness as part of standard clinical care, particularly for individuals with depression. Even in the absence of formalised interventions, clinicians can adopt a proactive and curious stance by engaging patients in open conversations about loneliness and collaboratively identifying meaningful actions, such as community engagement, peer support, or social prescribing options. Given that loneliness is both common and modifiable, it warrants greater attention in treatment planning and offers an important opportunity to enhance mental health outcomes.

## Conclusions

This systematic review and meta-analysis found consistent longitudinal evidence that loneliness is associated with subsequent poorer depression outcomes within CMD samples, particularly among individuals with depressive disorders. These associations were observed across clinical and non-clinical settings, and across diverse population groups, suggesting a robust and generalisable effect. In contrast, evidence for anxiety, suicidal ideation, and treatment outcomes was limited and mixed, highlighting important gaps in the current literature. Given the high prevalence of loneliness and its role in sustaining depression, there is a clear need for further research into interventions that address loneliness in people with CMDs. Targeting loneliness within mental health care may represent a promising strategy to improve clinical outcomes, particularly for individuals with depression, and should be prioritised in future research, policy, and practice.

## Supplementary information

Additional file 1: Appendices 1-7. Appendix 1: Prisma Checklist. Appendix 2: Full search strategy. Appendix 3: Screening guides. Appendix 4: Newcastle–Ottawa Scale (NOS) and Table S1. NOS ratings by study. Appendix 5: Table S2. GRADE Certainty of evidence for each outcome. Appendix 6: Near misses Tables S3 and S4. Appendix 7: Figure S1. Forest plot of the exploratory combined meta-analysis.

## Data Availability

All data generated or analysed during this study are included in this published article and its supplementary information files.

## Abbreviations

ACT: Acceptance and Commitment Therapy
BDI-II: Beck Depression Inventory-II
CBT: Cognitive Behavioural Therapy
CES-D-10: 10-item Center for Epidemiologic Studies Depression Scale
CI: Confidence Interval
CIDI: Composite International Diagnostic Interview
CLSA: Canadian Longitudinal Study on Aging
CMDs: Common mental disorders
CORE Unit: Centre for Outcomes Research and Effectiveness
DBT: Dialectical Behaviour Therapy
GAD: Generalised anxiety disorder
GAD-7: Generalized Anxiety Disorder-7
GRADE: Grading of Recommendations Assessment, Development and Evaluations
HR: Hazard ratio
IDS: Inventory of Depressive Symptomatology
IRR: Incidence rate ratio
MDD: Major depressive disorder
NESDA: Netherlands Study of Depression and Anxiety
NESDO: Netherlands Study of Depression in Older Persons
NICE: National Institute for Health and Care Excellence
NLP: Natural language processing
NOS: Newcastle–Ottawa Scale
OCD: Obsessive-compulsive disorder
OR: Odds ratio
PHQ-9: Patient Health Questionnaire-9
PRISMA: Preferred Reporting Items for Systematic Reviews and Meta-Analyses
PROMIS: Patient-Reported Outcomes Measurement Information System
PROSPERO: International Prospective Register of Systematic Reviews
PTSD: Post-traumatic stress disorder
RCTs: Randomised controlled trials
REML: Restricted maximum likelihood
RRR: Relative risk ratio
SIDAS: Suicidal Ideation Attributes Scale
